# Critical behavior and the Kibble-Zurek mechanism in a musical phase transition

**DOI:** 10.1371/journal.pone.0280227

**Published:** 2023-01-23

**Authors:** Huay Din, Jesse Berezovsky

**Affiliations:** Department of Physics, Case Western Reserve University, Cleveland, Ohio, United States of America; Coventry University, UNITED KINGDOM

## Abstract

We investigate the critical phenomena emerging from a statistical mechanics model of musical harmony on a three-dimensional (3D) lattice, and the resulting structure of the ordered phase. In this model, each lattice site represents a tone, with nearest neighbors interacting via the perception of dissonance between them. With dissonance assumed to be an octave-wise periodic function of pitch difference, this model is a 3D XY system with the same symmetry and dimensionality as superfluid helium and models of the cosmological axion field. We use numerical simulation to observe a phase transition from disordered sound to ordered arrangements of musical pitches as a parameter analogous to the temperature is quenched towards zero. We observe the divergence of correlation length and relaxation time at the phase boundary, consistent with the critical exponents in similar systems. Furthermore, the quenched low-temperature phase of these systems displays topological defects in the form of vortex strings that thread throughout the system volume. We observe the formation of these vortex strings in accordance with the Kibble-Zurek mechanism, and discuss the structure of these vortex strings in the context of the theory of musical harmony, finding both similarities to established music theory, and uncovering new avenues to explore.

## Introduction

Disparate thermodynamic systems, with only symmetry and dimensionality in common, are linked by universal critical behavior near the boundaries between phases. We have previously proposed that musical harmony can be described as a thermodynamic system of tones interacting via the perception of dissonance [1], and shown that phase transitions occur between disordered sound and ordered arrangements of pitches as used in existing musical systems. Here, we explore the behavior of this XY-like system on a three-dimensional lattice, and gain understanding by comparing to the behavior of similar 3D XY systems such as superfluid helium, easy-plane magnets, and the hypothesized cosmological axion field.

Near a phase transition, quantities such as the energy relaxation time and correlation length are expected to diverge with power law behavior described by critical exponents. Here, we will see that the relaxation time for dissonance between tones and the correlation length between musical pitches in this model diverge near the phase transition between disordered sound and music in a way consistent with the critical exponents in similar systems.

A notable feature of 3D XY systems is that they support defects in the form of vortex strings, which, as described by the Kibble-Zurek Mechanism (KZM), may become frozen into the low-temperature phase following a quench through the transition [2]. Evidence of these vortices and vortex strings has been observed in condensed matter systems [3–15], and are expected to manifest in the axion field as a network of “cosmic strings” threading throughout the universe [16, 17]. Interestingly, the KZM has been considered previously as a qualitative metaphor for structures in musical compositions [18]. Here, we observe these vortex strings in a simulation of a quenched system of interacting tones, with the expected dependence on quench rate.

In previous work, we postulated that a system of musical harmony tends to minimize dissonant sounds, while also tending to maximize the variety of potential compositions [1]. By making an analogy to the tendency of a physical system in the canonical ensemble to both minimize energy and maximize entropy, we can construct a “free energy” in which the trade-off between dissonance and compositional variety is set by a “temperature” *T*. We can then apply any methods of statistical mechanics based on free energy minimization. However, the analogy does not extend in the other direction. The underlying reason for the trade-off between internal energy and entropy is not the same as that between dissonance and musical complexity. In the former case, the reason is the combination of conservation of total energy and maximization of total entropy in the system and reservoir. In the latter case, psychological factors are at play. As such, the analogy does not extend to the more general results of thermodynamics. We used the mean field approximation to show that interacting tones spontaneously order into discrete sets of pitches, including the 12-fold division of an octave used in Western music, as well as other octave divisions used in non-Western systems of music.

The previously studied mean field model [1] considers a system of tones in which each tone interacts with all other tones. This approximation is unrealistic, in that in typical music at most several distinct pitch classes are sounded together as a chord. Moreover, certain pitch classes are more likely to be sounded together than others. If we instead consider a system of tones on a lattice that only interact with a finite set of neighbors, we can more realistically model the structure of musical harmony, and allow these short range correlations to emerge. This lattice model will also allow us to compare to traditional descriptions of Western harmony that are based on graphs or lattices such as the Tonnetz [19] or the Fokker lattice [20]. The dynamics of this model reflect the development of a system of musical harmony. Within a given culture, composers and musicians experiment with new pitches and new combinations of pitches. Our model posits that this experimentation is driven by the competing desires for consonant combinations of sounds, and sufficient variety to allow for novelty and a wide range of artistic expression. We will see below that the dynamics of our model are captured both by continuous adjustments of pitch, representing gradual tuning, and by discontinuous jumps of pitch, representing trying out wholly new pitches or new combinations of pitches. Though we cannot hope to quantitatively capture the dynamics of such a cultural process, the state of the system after quenching through the phase transition bears striking resemblance to existing systems of musical harmony. In previous work [1], we used numerical simulations of a 2D lattice of tones to observe a Kosterlitz-Thouless transition to a low-temperature state characterized by vortices of pitch. These vortices were interpreted as musical chords.

Here, we extend this result into three dimensions, and find that the resulting vortex strings provide a new window into musical harmony, and suggest new musical ideas to explore. With the vortices representing chords, the vortex strings can be interpreted as chord progressions. We find that the chords and chord progressions thus described mirror those often found in established Western music theory. These results also offer the possibility of adjusting the inputs to the model to rationally generate new systems of musical harmony for the basis of human or algorithmic composition.

### XY model of musical harmony

An XY model describes a system with O(2) symmetry, with a two-dimensional unit vector ${\vec{s}}_{i}$ on every site determined by an angle *θ*. In some cases, ${\vec{s}}_{i}$ represents a vector in space, such as the magnetization of an easy-plane ferromagnet [21]. In other cases, ${\vec{s}}_{i}$ represents some other periodic quantity, such as the phase of a superfluid wave function [22], or the phase of the hypothesized axion field [16].

In this work, the vector ${\vec{s}}_{i}$ represents musical pitch. Pitch is specified by the fundamental frequency *f* of a tone. On a logarithmic scale, the perception of pitch exhibits periodicity with *f*. Specifically, tones with frequencies that differ by a factor of two (i.e. an octave) are perceived to belong to the same “pitch class.” Instead of an angle *θ* with period of 2*π*, $\vec{s_{i}}$ here are specified by logarithmic frequency *p* = log_2_(*f*/*f*_0_) with period 1, where *f*_0_ is an arbitrary constant.

**Fig 1:**
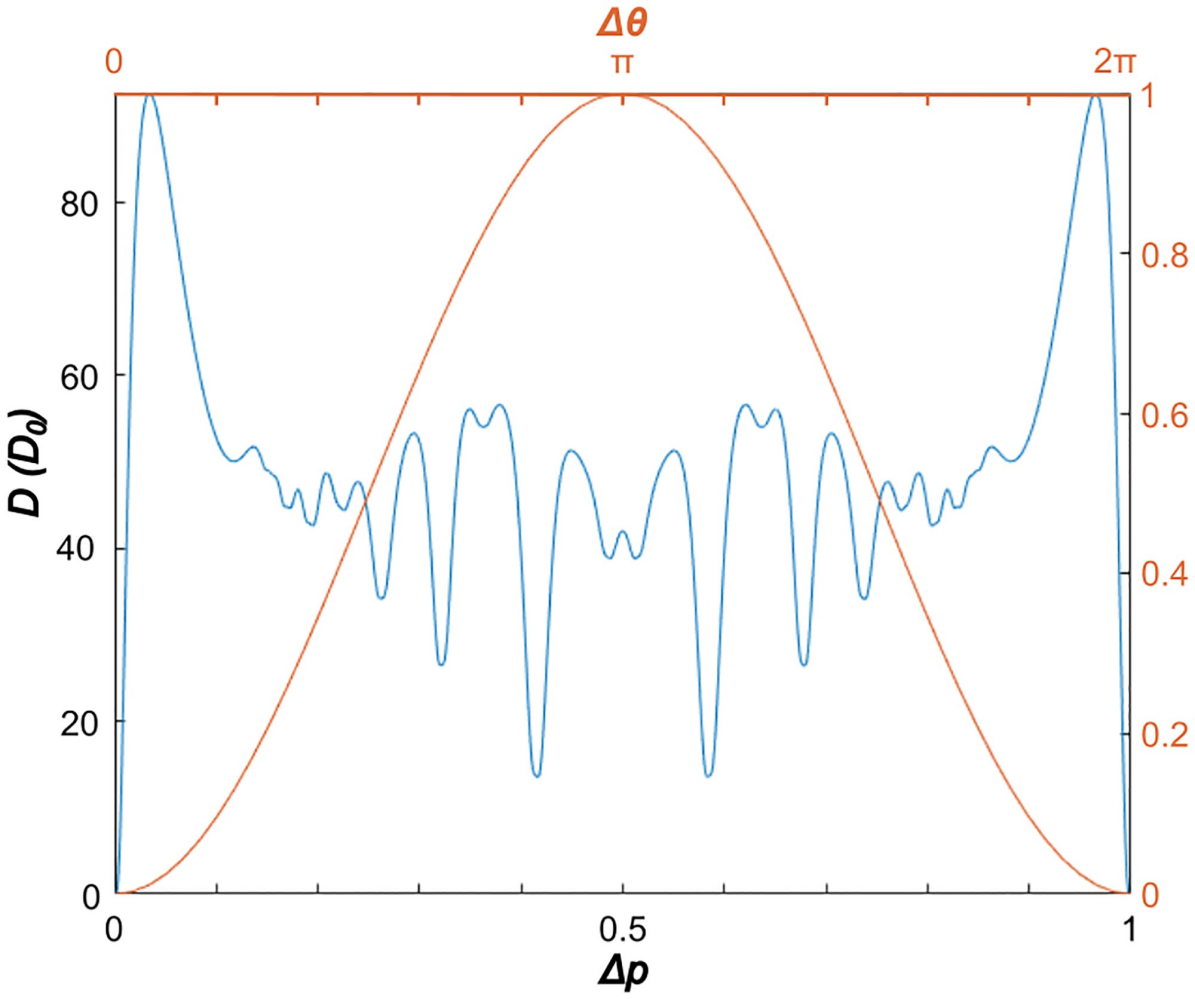
XY interactions. Dissonance function *D* (blue) as a function of logarithmic pitch difference Δ*p* over one octave, used here as the interaction between neighboring tones. For comparison, a more typical interaction energy ∝(1 − cos(Δ*θ*)) is shown in red.

In typical XY models, the interaction energy is often described by $u_{i,j}=J{\vec{s}}_{i}\cdot {\vec{s}}_{j}=J\text{cos}\left(\theta_{i}-\theta_{j}\right)$ with *J* < 0, as illustrated by the red curve in Fig 1. With a minimum *u* at Δ*θ* = 0, the ground state of the system is described by all ${\vec{s}}_{i}$ aligned, with an arbitrary direction chosen by spontaneous symmetry breaking. In general, small angles between interacting sites are energetically favored.

In the model here, the interaction between tones is described by the perceived dissonance *D*(Δ*p*) between those tones, depending only on the pitch difference Δ*p* = |*p*_*i*_−*p*_*j*_|. Experimentally, the perception of dissonance has been found to subtly depend on many factors, such as culture [23], musical training [24], and expectation [25]. There are, however, some underlying commonalities. Closely-spaced (but not identical) pitches are almost always perceived to be rough or unpleasant [26]. Intervals with fundamental frequencies in small integer ratios (e.g. 2: 1 or 3: 2) are typically found to be consonant, and intervals slightly deviating from these ratios are found to be dissonant. Neurological experiments in both humans and animals have shown that these effects are partially innate, and partially acquired by musical training or general exposure [27]. So though we expect *D*(Δ*p*) to be somewhat different between different cultures, and even between different people, we expect the prominent maxima and minima of *D*(Δ*p*) to appear in the same places. As such, the mechanisms of dissonance perception will be neglected here, and we will follow a simple approximation that yields a reasonable *D*(Δ*p*).

The dissonance function *D*(Δ*p*) can be quantified based on empirical psycho-acoustical measurements, and the spectrum of the waveform of the tones [1, 28–30]. The spectrum of a tone can be approximated by a set of “partials”: sinusoidal components at the fundamental frequency *f*, and at some set of other frequencies related to the fundamental. In a simple approximation, dissonance arises from closely spaced, but not identical, frequency components [29]. Here, we take the prototypical case of a sawtooth waveform, in which partials have amplitudes proportional to *α*_*n*_ = 1/*n* and frequencies proportional to *φ*_*n*_ = *n*, with *n* = 1, 2, 3…. In general, *φ*_*n*_ and *α*_*n*_ determine the timbre of the sound. Summing the contributions of all pairs of partials in each tone and imposing the octave-wise periodicity yields a periodic *D*(Δ*p*) in the range Δ*p* = [0, 1). (See the Methods section below for more detail.)

Fig 1 shows *D*(Δ*p*) (blue) in comparison to the typical cosine interaction energy. *D*(Δ*p*) is plotted in units of an arbitrary constant *D*_0_, which is consistently used here for both dissonance and temperature. Like the typical interaction energy, *D* has a minimum at Δ*p* = 0 indicating that the ground state of this system will also be with all ${\vec{s}}_{i}$ aligned, or in other words, a single pitch class. Unlike the typical case, however, *D*(Δ*p*) has maxima at small deviations from Δ*p* = 0 and many other local minima in between. This will result in more complex behavior away from the ground state.

Other XY systems have been explored with multiple minima in the interaction energy, such as ferroelectric ordering in hexagonal manganites [6] or stacking order in bilayer graphene [5]. In those cases, sixfold symmetry emerges due to the underlying crystal structure. In contrast, the multiple minima in the interaction between tones are intrinsic to the dissonance function, and are not imposed by any underlying structure. Moreover, the minima in *D* do not occur at positions commensurate with the period. For example, the two second-lowest minima in either side of Δ*p* = 0.5 arise from the dissonance between tones with fundamental frequencies in the ratio of 3/2 and 4/3, and therefore occur at Δ*p* = log_2_3/2 ≈ 0.585 and Δ*p* = log_2_4/3 ≈ 0.415. In music, pairs of tones with these pitch differences are known as intervals of a perfect fifth and a perfect fourth, respectively. These, and other significant minima in *D*, lead to the consonant intervals traditionally used in music.

The multiple minima in *D* result in multiple order parameters $M_{k}=\langle e^{2\pi ikp_{j}}\rangle$, for *k* = 1, 2… where brackets indicate averaging over all sites *j*. In contrast, the order parameter for the typical cosine interaction energy can be characterized by *M*_1_ only. The amplitude and phase of *M*_*k*_ correspond to the amplitude and phase of the *k*-fold periodic ordering of pitches in an octave. These order parameters are similar to those that arise in the study of bond orientational order [31, 32].

XY systems often manifest topological defects in the form of vortices. In 2D, a vortex forms around a zero-dimensional point where the surrounding values rotate around the center with nonzero winding number. These defects extend into a 3D XY model as one-dimensional vortex strings, in which the vortex core follows a path through space. In standard XY models with interaction energy ${\vec{s}}_{i}\cdot {\vec{s}}_{j}$, energy is minimized by rotating the angle around the vortex as gradually as possible. When there are multiple minima in the interaction energy, it may be favorable for the angle to undergo sudden jumps around the vortex [5, 6]. This results in domains of largely constant ${\vec{s}}_{i}$ separated by domain walls. A vortex forms when three or more domains meet at a point. We expect this type of vortex to form in the model here, given the multiple local minima in *D*. As we will see below, these vortices can be interpreted as chords with three or more pitches with mutual consonance between them.

### Critical behavior and the Kibble-Zurek mechanism

In general, a phase transition occurs when a thermodynamic system passes between a higher-symmetry macrostate and a lower-symmetry macrostate, with spontaneous symmetry breaking occurring at the phase boundary. Approaching that boundary, the system undergoes critical behavior in which macroscopic observables are described by power law behavior with universal exponents depending only on the symmetry of the system. In the model of music here, the high-temperature disordered phase corresponds to random sonic noise with no long range order and complete symmetry, whereas the ground state corresponds to a single pitch class with infinite-range order and no symmetry other than the assumed octave periodicity.

We will explore the process by which the system transitions from disordered sound to ordered musical pitch. Important here is the fact that among the critical power-law-dependent observables is the energy (or dissonance) relaxation time, which diverges at the phase boundary. As such, if the temperature is lowered through the phase transition at any finite quench rate, the system must be out of equilibrium in some range about the phase boundary. In XY systems, this nonequilibrium passage results in vortices or vortex strings frozen into the low temperature state via the KZM, and analysis of this critical behavior allows us to predict how the typical concentration of defects depends on the quench rate.

The macroscopic observables of interest here are the correlation length *ξ* and relaxation time *τ*. As temperature approaches the critical temperature *T*_*c*_, those parameters diverge with power law behavior in $\varepsilon \left(T\right)=\frac{T-T_{c}}{T_{c}}$. Specifically,

$$\begin{array}{c} \xi \left(\varepsilon \right)=\frac{\xi_{0}}{{\left|\varepsilon \right|}^{\nu }} \end{array}$$ (1)

and

$$\begin{array}{c} \tau \left(\varepsilon \right)=\frac{\tau_{0}}{{\left|\varepsilon \right|}^{z\nu }}. \end{array}$$ (2)

Above, *ξ*_0_ and *τ*_0_ are dimensionful constants that depend on the microphysics, and *ν* and *z* are the correlation length critical exponent and dynamic critical exponent respectively.

The KZM describes defect formation for finite quench rates. The speed of a quench through the transition determines how well the system is able to equilibrate near the critical point, where *τ* diverges. The quench rate $\tau_{Q}^{-1}$ specifies the time-dependence of the temperature: *ε*(*t*) = −*t*/*τ*_*Q*_. In an XY system, the disordered phase can be viewed as a collection of vortices and antivortices that can be created and annihilated, keeping the total topological charge constant. Assuming a total topological charge of zero, all defects should annihilate upon undergoing an equilibrium phase transition to the low-temperature phase. However, because it is impossible in practice to remain in equilibrium near the critical point, defects become “frozen” into the system. The freeze-in process begins at a temperature

$$\begin{array}{c} \hat{\varepsilon }={\left(\frac{\tau_{0}}{\tau_{Q}}\right)}^{\frac{1}{1+z\nu }} \end{array}$$ (3)

at which *τ* exceeds the time remaining to reach *ε* = 0. Assuming symmetric behavior about *ε* = 0, at $\varepsilon <-\hat{\varepsilon }$ the system can now locally relax to equilibrium, but sufficiently spaced vortices will not be able to relax. In a 3D XY system, this process results in a network of vortex strings that persist as *T* goes to zero.

Because of the inability to reach equilibrium, the correlation length *ξ* does not diverge at *ε* = 0, but instead pauses at an approximately constant value

$$\begin{array}{c} \hat{\xi }=\xi_{0}{\left(\frac{\tau_{Q}}{\tau_{0}}\right)}^{\frac{\nu }{1+z\nu }}, \end{array}$$ (4)

before beginning to increase again at $\varepsilon <-\hat{\varepsilon }$.

The concentration of or typical spacing between vortex strings frozen in as *T* → 0 is directly related to $\hat{\xi }$. At very slow quench rate (large *τ*_*Q*_), $\hat{\xi }$ becomes large, approaching the equilibrium result of *ξ* → ∞, and vortex strings become increasingly sparse. For a fast quench (*τ*_*Q*_ small), the spacing between vortex strings is smaller. In experiments and simulations in other systems, the predicted critical behavior of the 3D XY model has been observed, with defect concentration roughly in agreement with the predicted $\hat{\xi }$, typically within an order of magnitude [4, 6–15, 33, 34], though precise confirmation is complicated by the need to avoid other mechanisms of defect formation and the need to quantitatively parameterize the dynamics of vorticity generation [35].

## Simulations

We performed numerical simulations to study the behavior of a 3D lattice of tones upon quenching through the phase transition. We extract *ξ* and *τ* as a function of *T* by fitting simulation results and then we compare the observed critical behavior to that of similar systems.

We used a combination of Metropolis Monte Carlo (MMC) and Langevin dynamics to simulate this 3D XY system in MATLAB. The MMC and Langevin dynamics served to sample random configurations and to explore to local configuration space, respectively. Each MMC step selects one site at random and accepts a randomly-selected change in pitch depending on how that change affects the change in total dissonance, relative to the temperature. Performing many such MMC steps allows the simulation to explore the entire configuration space of the pitches on the lattice. The Langevin dynamics algorithm numerically solves the Langevin equation, where the dissonance function takes the place of the potential energy. These dynamics describe continuous changes in pitch that tend to minimize dissonance with the addition of thermal noise. When the MMC steps place the system near a local minimum in configuration space, the Langevin dynamics are more efficient at exploring that local region. Musically, the Langevin dynamics can be viewed as small adjustments of intonation made when tuning or playing an instrument, whereas the MMC steps represent adding or subtracting new pitches or chords to the system of harmony. More details of the simulation technique are given in [1], in the Methods section below, and in the S1 Text.

The 3D cubic lattice of 40 × 40 × 40 tones was initialized with a random pitch distribution in the high symmetry phase (*ε* > 0). The dissonance was calculated among the nearest six neighbors with periodic boundary conditions. For each value of *T*, the MMC-Langevin algorithms were alternated for a predetermined number of steps. The quench rate, $\tau_{Q}^{-1}$, was controlled by setting the number of steps taken at each temperature, and the size of the temperature steps. Values of *τ*_*Q*_ are in units of simulation time *t*_0_ defined in the Methods section below, and values of *T* are in units of *D*_0_, the same as the units of *D* in Fig 1. Both the values of *D*_0_ and *t*_0_ are arbitrary, in that they do not correspond to physically measurable quantities. Nevertheless, we can use these internally consistent units to test the predictions of the KZM.

**Fig 2:**
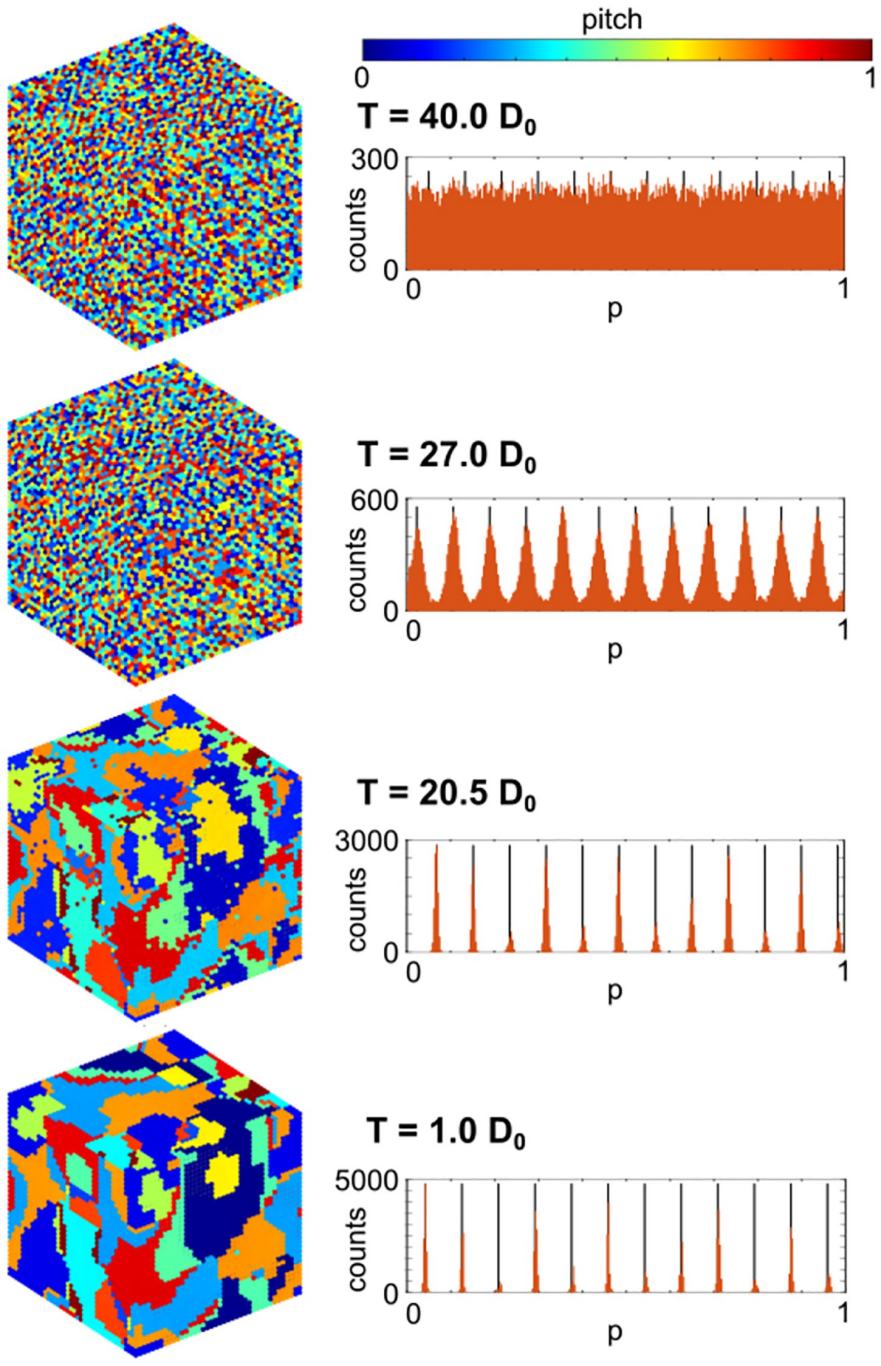
Typical simulation results. Right: Histograms of pitches *p* over one octave on the simulated lattice, as temperature is quenched at *τ*_*Q*_ = 3276*t*_0_ from above *T*_*c*_ at *T* = 40*D*_0_ to near *T*_*c*_ ≈ 27*D*_0_, and then towards *T* = 0. The 12-fold octave division used in Western music emerges near *T* = *T*_*c*_ illustrated by the 12 equally spaced black lines. Left: Simulated lattice of tones at the same simulation steps shown at right. Domains of nearly constant pitch emerge at low temperature, separated by discrete domain walls.

Typical simulation results are shown in Fig 2, for *τ*_*Q*_ = 3276*t*_0_. At the left, the lattice of tones is plotted with the pitch value *p* represented by color. Four snapshots are shown as the temperature is lowered from the initial value of *T* = 40*D*_0_ down to low temperature at *T* = *D*_0_. At the right, histograms are shown of the pitch values present on the lattice at each *T* shown. At *T* = 40*D*_0_, we see a uniformly random distribution of pitches in the histogram, and essentially no correlations visible between neighboring tones on the lattice. When the temperature is lowered to *T* = 27*D*_0_ ≈ *T*_*c*_, there are still no correlations clearly visible to the eye, but we can see order beginning to emerge in the histogram. Specifically, 12 peaks have emerged in the pitch histogram, reflecting the 12 pitches per octave used in Western music. As the temperature is lowered further to *T* = 20.5*D*_0_, the peaks in the histogram have sharpened further into distinctly separated pitches, and the small domains of constant pitch are now visible on the lattice. As the temperature is lowered to near zero (*T* = *D*_0_), the domains of constant pitch have saturated to near their final size—the remaining disorder is frozen in. Video of this simulation run is shown in S3 Video, and two other runs with different *τ*_*Q*_ are shown in S1 and S5 Videos.

## Critical behavior

We will now analyze the critical behavior present in the simulation results. We first extract the critical temperature *T*_*c*_, and values of *τ* and *ξ* as a function of *ε*. We fit to obtain values for the critical exponents *ν* and *z*, and the scaling values *τ*_0_ and *ξ*_0_. With these values determined, we can find the $\hat{\xi }$ predicted by the KZM, and compare to the computationally measured $\hat{\xi }$ values, finding good agreement.

**Fig 3:**
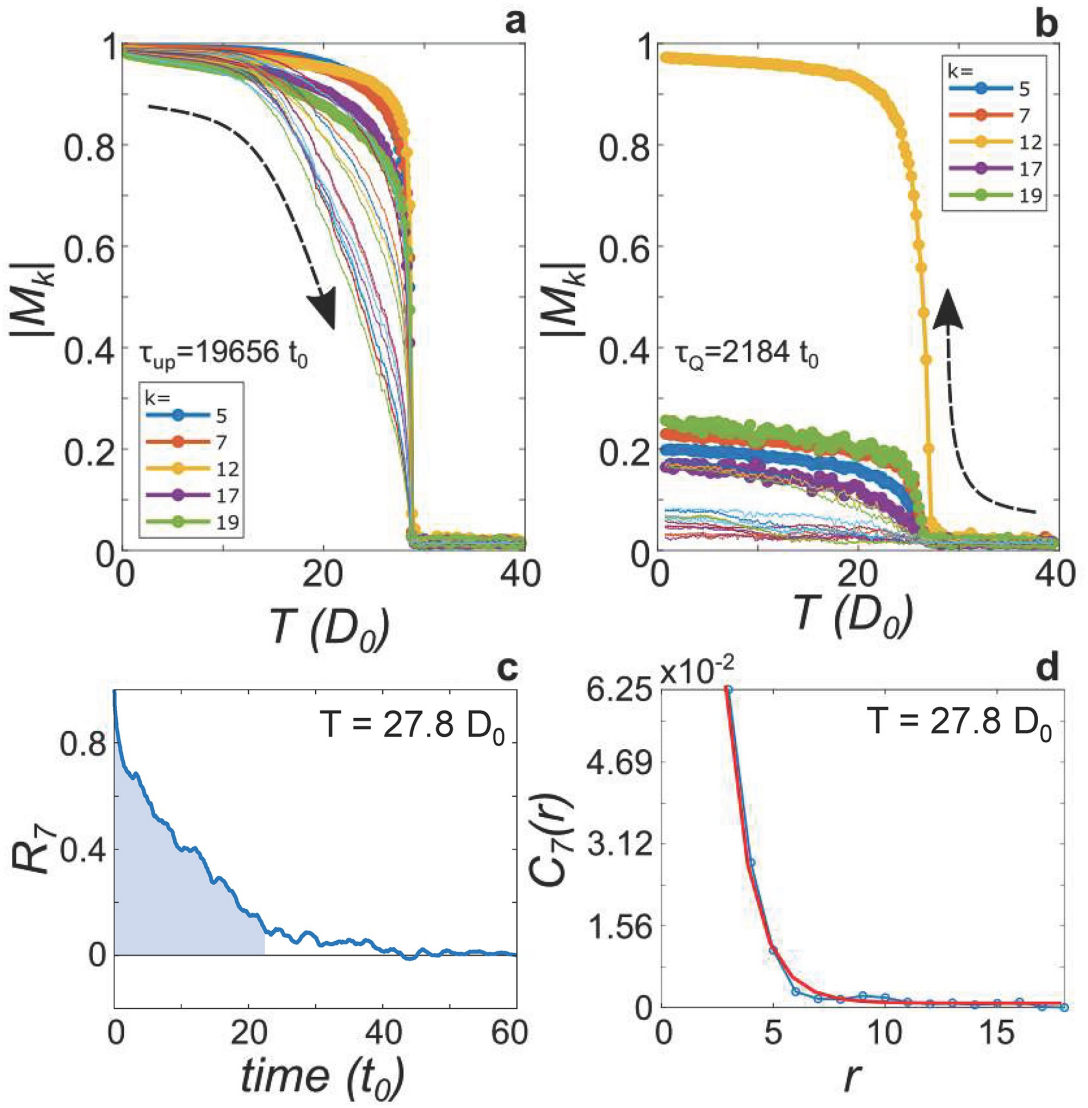
Parameter extraction. (a) Magnitude of order parameters |*M*_*k*_| vs. *T* for a slow temperature sweep up, showing *k* = 1 − 20. Prominent values of *k* are indicated in legend. (b) Same as (a) but for a faster quench down. (c) Example of determination of one value of *τ* as the area of the shaded region under the curve of the magnitude of the autocorrelation *R*_7_(*t*) of the *M*_7_ order parameter over time until *R*_7_ first crosses 0.1. (d) Example of determination of one value of *ξ* by performing an exponential fit to the correlation function *C*_7_(*r*). Due the finite system and simulation time, both *R*_*k*_ and *C*_*k*_ have small imaginary parts that we neglect.

The phase transition is clearly seen by plotting the order parameters *M*_*k*_ vs. *T*. Fig 3(a) shows |*M*_*k*_| as *T* is swept from *D*_0_ upwards relatively slowly (*τ*_*up*_ = 19, 656*t*_0_). Values of *k* from 1 to 20 are shown, with the most prominent values indicated in the legend. The simulation is initialized into the expected ground state with all pitches equal, and where all |*M*_*k*_| = 1. As *T* increases, all |*M*_*k*_| begin to decrease, as the pitch distribution begins to spread out within one local minimum of dissonance, or jump to other minima. Approaching *T*_*c*_, all |*M*_*k*_| fall abruptly to zero, with Curie-Weiss-like behavior. Above *T*_*c*_, all *M*_*k*_ = 0.

Fig 3(b) shows |*M*_*k*_| as *T* is quenched from *T* = 40*D*_0_ down in a relatively faster sweep (*τ*_*Q*_ = 2184*t*_0_). The simulation is initialized with uniformly random pitches which rapidly relax to equilibrium (the relaxation time *τ* < *t*_0_ at this temperature, as shown below). As expected, all |*M*_*k*_| = 0 at high temperature, with a sharp increase at *T*_*c*_. Similar to the sweep up, |*M*_12_| increases towards 1 as *T* goes to zero. The other |*M*_*k*_| shown, however, saturate at lower values because the rapid quench precludes the system from reaching equilibrium, as described by the KZM. We identify *T*_*c*_ from the position of the kink in *M*_*k*_. There is a slight difference (≈0.5*D*_0_) in *T*_*c*_ for the *M*_12_ order parameter and for all other *M*_*k*_. This can be understood as a coupled mode transition, in which the *M*_12_ order parameter destabilizes first, and then coupling to the other order parameters drives the rest of the modes to destabilize. This behavior in the mean field case is discussed in Ref. [1], and the KZM has been studied in such a system in Ref. [36]. The kink in the sweep up is observed slightly higher (by 1.95*D*_0_) than in the sweep down. This may indicate that the transition is weakly first order. Though *τ* is not expected to diverge in a first order transition, it may still become large enough for the KZM description to hold [7, 33]. Another model system that is weakly first order is the 3D 3-state Potts model [37, 38]. In this model, each site can take on one of three states. Interacting sites in the same state have low energy, and interacting sites in different states have a higher energy. This bears some similarity to the system studied here, in that the interaction potential *D*(Δ*p*) has one global minimum, and two next-deepest minima. See S1 Text for further simulations and discussion regarding the nature of the phase transition. For the purposes of studying critical behavior of the *M*_7_ order parameter during a quench, we take *T*_*c*_ = 27.3*D*_0_ from the quench shown in Fig 3(b). All other simulated quenches show *T*_*c*_ at this value.

We next extract *τ* from the autocorrelation *R*_*k*_ of the order parameter *M*_*k*_ as it fluctuates in time, and *ξ* by fitting the correlation function to *C*(*r*)∝exp(−*r*/*ξ*). The simulation proceeds by performing a fixed number of MMC-Langevin steps at a constant temperature, then stepping to the next temperature point. At each fixed temperature, the order parameters *M*_*k*_ fluctuate about their equilibrium value (which is zero for *T* > *T*_*c*_). According to the fluctuation-dissipation theorem, the autocorrelation of these fluctuations in time decays with time constant *τ*, as demonstrated in Fig 3(c). A value of the relaxation time *τ* is obtained as the numerical integral of *R*_*k*_(*t*)/*R*_*k*_(0) from *t* = 0 to *t* = *t*_thresh_ at which *R*_*k*_(*t*_thresh_)/*R*_*k*_(0) = 0.1, as illustrated in Fig 3(c).

In general, the two-point correlation function *C*(*r*) measures how the order parameter at one site predicts the value at another site a distance *r* away. In a typical XY model, $C\left(r\right)={\langle {\vec{s}}_{i}\cdot {\vec{s}}_{j}\rangle }_{r}={\langle e^{i(\theta_{i}-\theta_{j})}\rangle }_{r}$, where 〈…〉_*r*_ means the average over sites separated by *r*, and the equality of the two expressions arises because of cancellation of the imaginary parts by symmetry. Here, we have a correlation function *C*_*k*_(*r*) = 〈*e*^2*πik*(*p*_*i*_ − *p*_*j*_)^〉_*r*_ associated with each order parameter *M*_*k*_. *C*_*k*_(*r*) then measures how *k*-fold order persists over a distance *r*. In the quenched state in Fig 2, we see that the entire volume displays the same 12-fold order, which indicates that *C*_12_(*r*) approaches a constant with *ξ* large compared to the system size at low *T*, as |*M*_12_|→1. That is, *ξ* for the *M*_12_ order parameter becomes large enough that no vortices in this order parameter remain frozen in as *T* → 0. Other *M*_*k*_, however, do not saturate to 1 after a quench, and the corresponding correlation functions *C*_*k*_(*r*) become saturated at a lower value, indicating that vortices become frozen in as *T* → 0. Here, we will focus on *C*_7_, which is associated with one of the largest |*M*_*k*_| that does not saturate to 1, and which therefore is expected to exhibit frozen-in vortices as *T* → 0. Given the overall 12-fold ordering, the *M*_*k*_ with *k* = 5, 7, 17, and 19 are similar because they all are equivalent to ±5 mod 12. See the supporting material in S1 Text for analysis of critical behavior for other values of *k*.

An example of *C*_7_(*r*) as calculated from the simulation is shown in Fig 3(d), where *r* is in units of lattice constant here and henceforth. Here, we have averaged 15 repetitions of the simulation to reduce fluctuations. In the 3D XY model, *C*(*r*) is expected to behave as an exponential decay in the long range limit [39]. We extract this long range decay constant *ξ* by performing an exponential fit, excluding the first three points, as shown in red.

**Fig 4:**
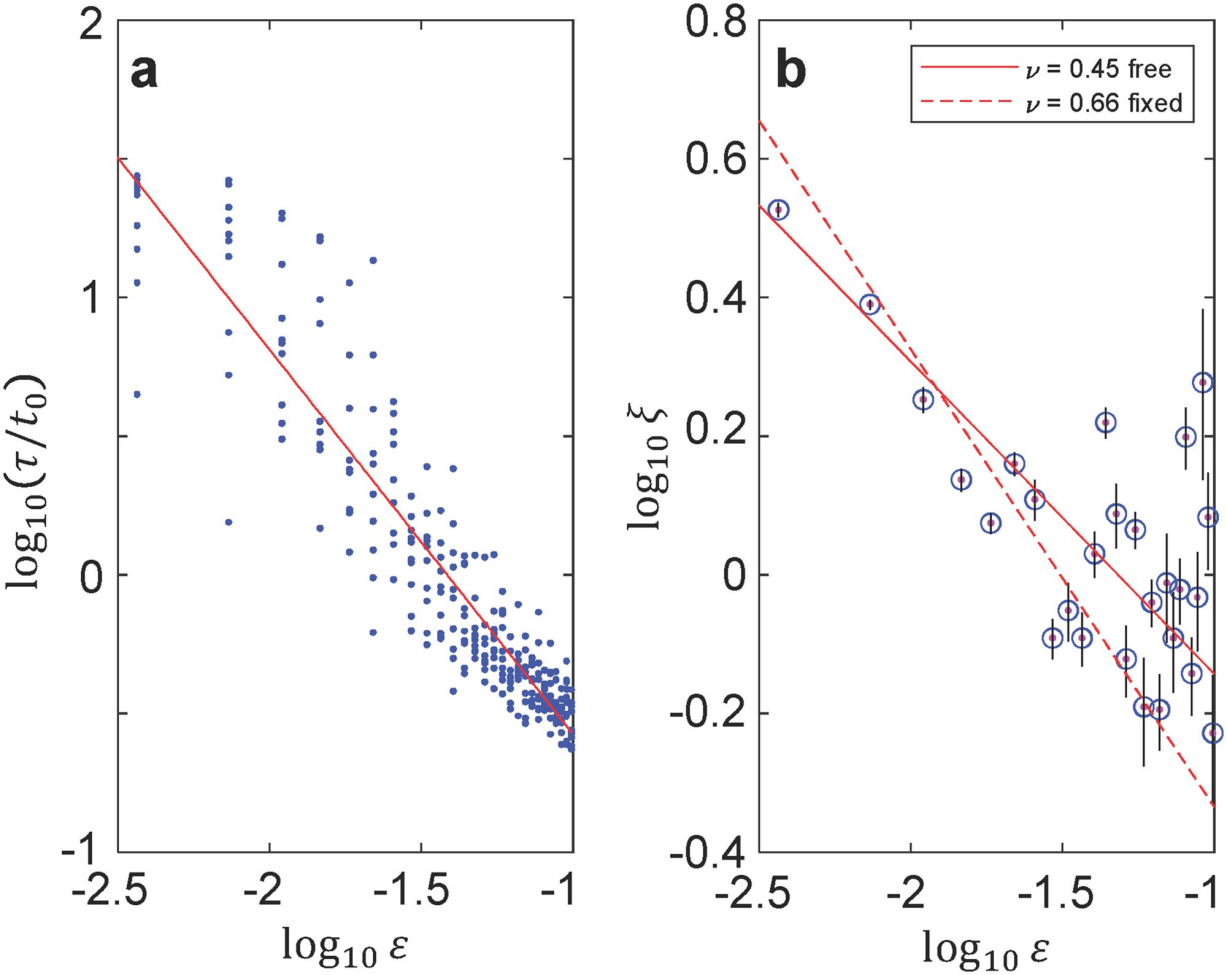
Fitting critical behavior. (a) *τ* vs. *ε* extracted from ten simulation runs with the same parameters on a log-log scale. Power law fit is shown in red, yielding *zν* = 1.39±0.03 and *τ*_0_ = (1.1±0.1) × 10^−2^*t*_0_. (b) Same as (a) but showing *ξ* vs. *ε* from 15 runs. At each *ε*, *C*_7_(*r*) was extracted from each run and averaged together before fitting to find *ξ*. Power law fits are shown in red, yielding *ν* = 0.45±0.04 and *ξ*_0_ = 0.25±0.05, with *ν* varied in the fit (solid line) or *ξ*_0_ = 0.101±0.005 with fixed *ν* = 0.66 (dashed line).

With values of *τ* and *ξ* vs. *T* extracted from multiple simulation runs at *τ*_*Q*_ = 16380 *t*_0_, we can observe the expected critical behavior and obtain values of *τ*_0_ and *ξ*_0_. We expect the trends of each variable to follow a power law near *ε* = 0 and *ε* > 0. (At *ε* < 0, the system is not in equilibrium.) We show the collected values of *τ* and *ξ* in Fig 4 on a log-log scale ((a) and (b), respectively). With a log-log scale, we can directly observe the critical exponent as the slope, and the dimensionful values as the intercepts. Away from *T*_*c*_, *τ* and *ξ* become too small to accurately fit, so we restrict our attention to 0 < *ε* < 0.1. The error in the values of *τ* can be seen from the spread of points at each temperature derived from ten repetitions of the simulated quench. The error bars on each value of *ξ* indicate the 1-*σ* confidence interval from the least-squares fit to the exponential decay function.

It was found that *τ* and *ξ* vs. *ε* are consistent with power law behavior, and exhibit critical exponents in the same range as similar well-studied systems. We performed linear least squares fits to the data on a log-log scale in order to obtain values of *ν*, *zν*, *τ*_0_, and *ξ*_0_. For the fit to *ξ*, we use the error bars *σ*_*i*_ to set weights $w_{i}=\sigma_{i}^{-2}$ for the *i*^*th*^ datapoint. For the fit to *τ*, the multiple repetitions at each temperature incorporate the uncertainty in the values into the fit. The fits, shown as solid red lines in Fig 4, yield critical exponents *ν* = 0.45±0.04 and *zν* = 1.39±0.03 and hence *z* = 3.1±0.3.

Because the values of *ξ* in Fig 4(b) have a significant scatter at larger values of *ε*, we use another method to determine *ν* exploiting finite size scaling [40, 41]. In a finite system of size *L*, such as the ones we simulate here, true critical behavior only emerges in the limit *L* → ∞. At a particular value of *L*, observable properties will not diverge, but will become peaked at a temperature *T*_*c*_(*L*) > *T*_*c*_, with *T*_*c*_(*L*) − *T*_*c*_ ∝ *L*^−1/*ν*^. We simulate quenches at *τ*_*Q*_ = 32760*t*_0_ on an *L* × *L* × *L* lattice, with *L* varying from *L* = 30 to *L* = 5. From these simulations, we obtain values of *T*_*c*_(*L*) and fit to obtain a value of *ν* = 0.66 ± 0.05, and hence *z* = 2.1 ± 0.2. The details of this analysis are given in the S1 Text Fixing this value of *ν* in the fit in Fig 4(b) yields the red dashed line, and a value of *ξ*_0_ = 0.101±0.005.

As discussed above, the model studied here is similar to both the XY model and the 3-state Potts model in three dimensions. We can then compare to the critical exponents in those systems. The 3D XY model has critical exponents *ν* = 0.67 and *zν* = 1.33 [39, 42], and the 3D 3-state Potts model has critical exponents *ν* = 0.5 and *zν* = 1.0 [38]. We see that the fitted exponents here are roughly in the same range. The value of *zν*, and the value of *ν* from finite size scaling are close to the 3D XY values, while the *ν* obtained from fitting *ξ* vs. *ε* is closer to the 3-state 3D Potts value. We note that the error bars on the fitted exponents come from the confidence interval of the fit result, and do not take into account possible systematic errors arising in the fitting to obtain values of *τ* and *ξ*. Notably, the values of *ξ* in Fig 4(b) at larger *ε* appear to skew higher. This may be caused by the difficulty of fitting *ξ* when the correlation length is less than one lattice site. This error may result in an underestimate of *ν* from direct fitting, and suggests that the finite-size scaling result of *ν* = 0.66 may be more accurate. It is also possible that the mode coupling between the different order parameters alters the critical behavior [36]. Given these complications, we can only conclude that *τ* and *ξ* display power law behavior with exponents in approximately the same range as these related systems.

With the values of *τ*_0_, *ξ*_0_, *ν* and *zν* we can calculate the predicted $\hat{\xi }$ and compare to the simulation. From the values extracted from Fig 4 and Eqs 3 and 4, we obtain ${\text{log}}_{10}\hat{\varepsilon }=-2.6$ and ${\text{log}}_{10}\hat{\xi }=0.56$ or ${\text{log}}_{10}\hat{\xi }=0.71$ for the fit with *ν* varied and fixed *ν* = 0.66, respectively. The value of $\hat{\varepsilon }$ confirms that the region shown in Fig 4 is in the adiabatic regime $\varepsilon >\hat{\varepsilon }$ where power-law behavior is expected. And in Fig 4(b), we can see that *ξ* is approaching these approximate values of $\hat{\xi }$ as *ε* approaches $\hat{\varepsilon }$.

**Fig 5:**
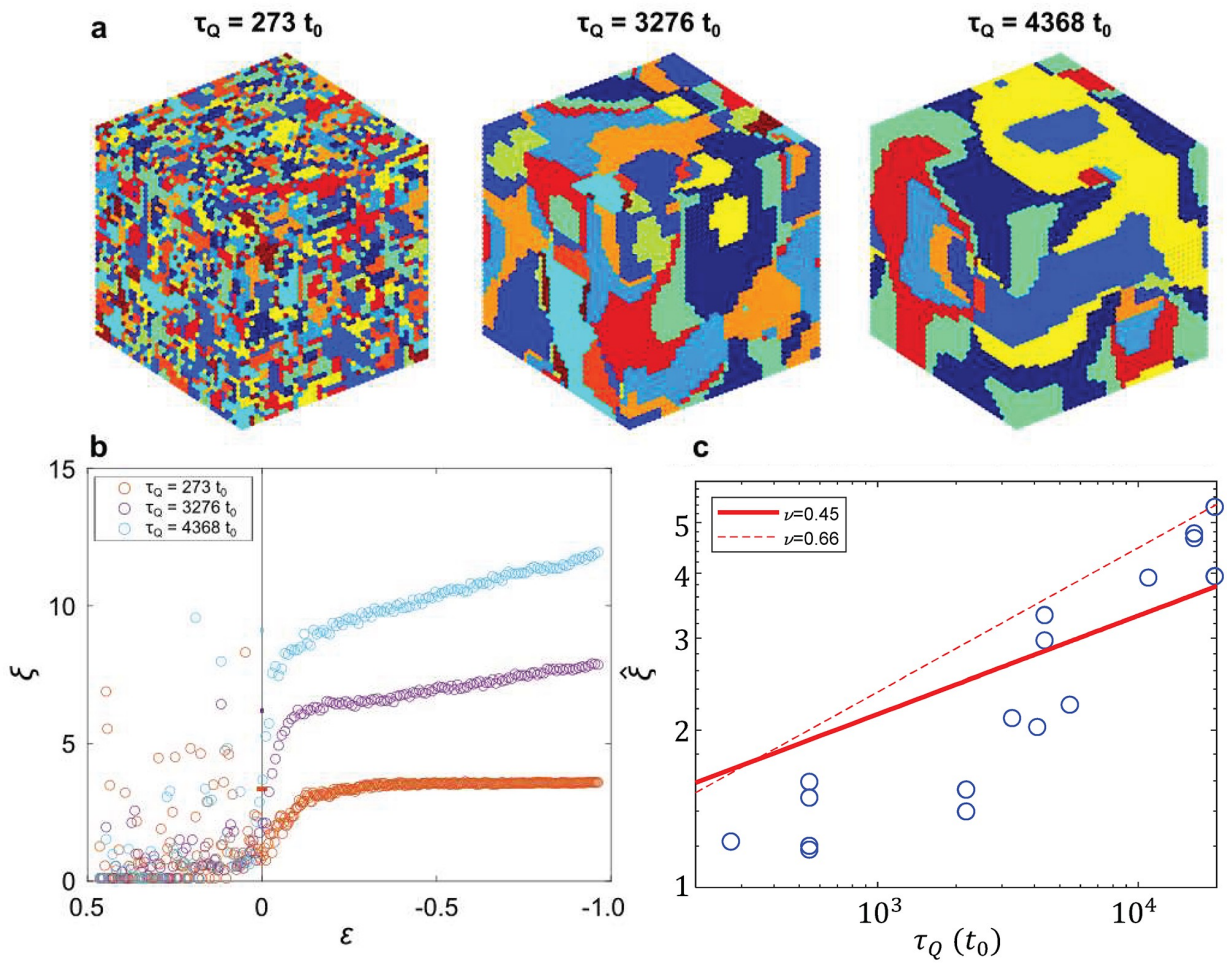
Comparison to KZM predictions. (a) Final simulation state at *T* = *D*_0_, following a quench at three *τ*_*Q*_. Color represents pitch *p* at each lattice site. As *τ*_*Q*_ increases, larger domain size, lower concentration of defects, and larger $\hat{\xi }$ can be seen by eye. (b) *ξ* vs. *ε* for the three simulation runs shown in (a), extracted by fitting to *C*_7_(*r*). (c) Values of $\hat{\xi }$ (blue points) extracted from simulation runs with different *τ*_*Q*_, compared to the prediction of the KZM (red).

To illustrate the dependence on *τ*_*Q*_, Fig 5(a) shows three simulation results at *T* = *D*_0_ following quenches at rates *τ*_*Q*_ = 273*t*_0_, 3276*t*_0_, and 4368*t*_0_. We can see by eye that larger *τ*_*Q*_ results in larger domain size, or equivalently, larger correlation length *ξ*(*T* → 0), or smaller concentration of frozen-in defects.

Quantitatively, Fig 5(b) shows *ξ*(*ε*) for the simulation runs shown in Fig 5(a). Note that the *ε* axis proceeds from high *ε* to low, in the direction of the quench. Above *T*_*c*_ (*ε* > 0), we can see the power law increase, which then reaches $\hat{\xi }$ as the dynamics become frozen at $\varepsilon =\hat{\varepsilon }$. As expected, the value of $\hat{\xi }$ increases as *τ*_*Q*_ increases. In each case, we indicate the “frozen” range $\pm \hat{\varepsilon }$ about *T*_*c*_ with a small colored bar, in which relaxation time *τ* exceeds the time until or past *T*_*c*_. These values range from $\hat{\varepsilon }\approx 0.005$ to 0.02. Though these ranges appear small on Fig 5(b), they last for significant simulation time ≈6*t*_0_—50*t*_0_. See S1 Text for a more detailed view of the frozen range $\pm \hat{\varepsilon }$. Below *T*_*c*_ ($\varepsilon <-\hat{\varepsilon }$), local fluctuations in the system can again equilibrate adiabatically, leading to further increase of *ξ*, but defects frozen in near *T*_*c*_ remain, leading to suppressed values of *ξ* for smaller *τ*_*Q*_.

Fig 5(c) shows the observed $\hat{\xi }$ values (blue circles) extracted from simulation runs with different *τ*_*Q*_, compared to the prediction of the KZM using the two fits for *ξ*_0_ with *ν* varied (solid red line) and fixed *ν* = 0.66 (dashed red line). We extract values of $\hat{\xi }$ as the mean of the *ξ* values in the frozen region between $\pm \hat{\varepsilon }$ (indicated by colored bars in Fig 5(b)). The red lines in Fig 5(c) are plots (not fits) of Eq 4, with the values of *τ*_0_, *ξ*_0_, *ν*, and *z* determined above in Fig 4. We see that our simulation results are in reasonable quantitative agreement with both the exponent and overall scale predicted by the KZM. The solid line overlaps more of the data points, but the dashed line (with *ν* obtained from finite size scaling) better matches the observed slope. This suggests that the exponent *ν* = 0.66 from finite size scaling is accurate, but the overestimate of $\hat{\xi }$ is again due to the scatter towards larger values of the measured *ξ* at larger *ε*. Similar results for the *M*_12_ order parameter are shown in S1 Text.

The results above have shown that in this model of musical harmony we observe a phase transition from disordered sound to ordered arrangements of twelve discrete pitches, and that defects appear in the quenched state in accordance with the KZM. In the next section, we describe the features of the quenched state, and compare them to the features of traditional Western harmony. First, we will give a brief introduction to the theory of musical harmony, then describe how the quenched state that we observe in our simulation relates to this theory. Specifically, we will see that the frozen-in vortices in the quenched state can be interpreted as common musical chords, with adjacent regions of these chords forming common musical scales. Further we will see that a path along the branching network of vortex strings can be interpreted as a possible chord progression, following a theory of chord progressions known as neo-Riemannian theory.

## Discussion

The theory of musical harmony focuses on which pitches are sounded together, or in close proximity, and how those harmonies are arranged in time. We have already seen above, and in Ref. [1], that the twelve pitch classes of Western music (e.g. A♭, A, B♭, B, C, D♭, D, E♭, E, F, G♭, G) emerge automatically in the quenched state of the model here. Below, we will refer to these pitch classes by integers 0–11. Next, we will review the foundations of traditional Western harmony, then discuss insights gained from the model presented here.

**Fig 6:**
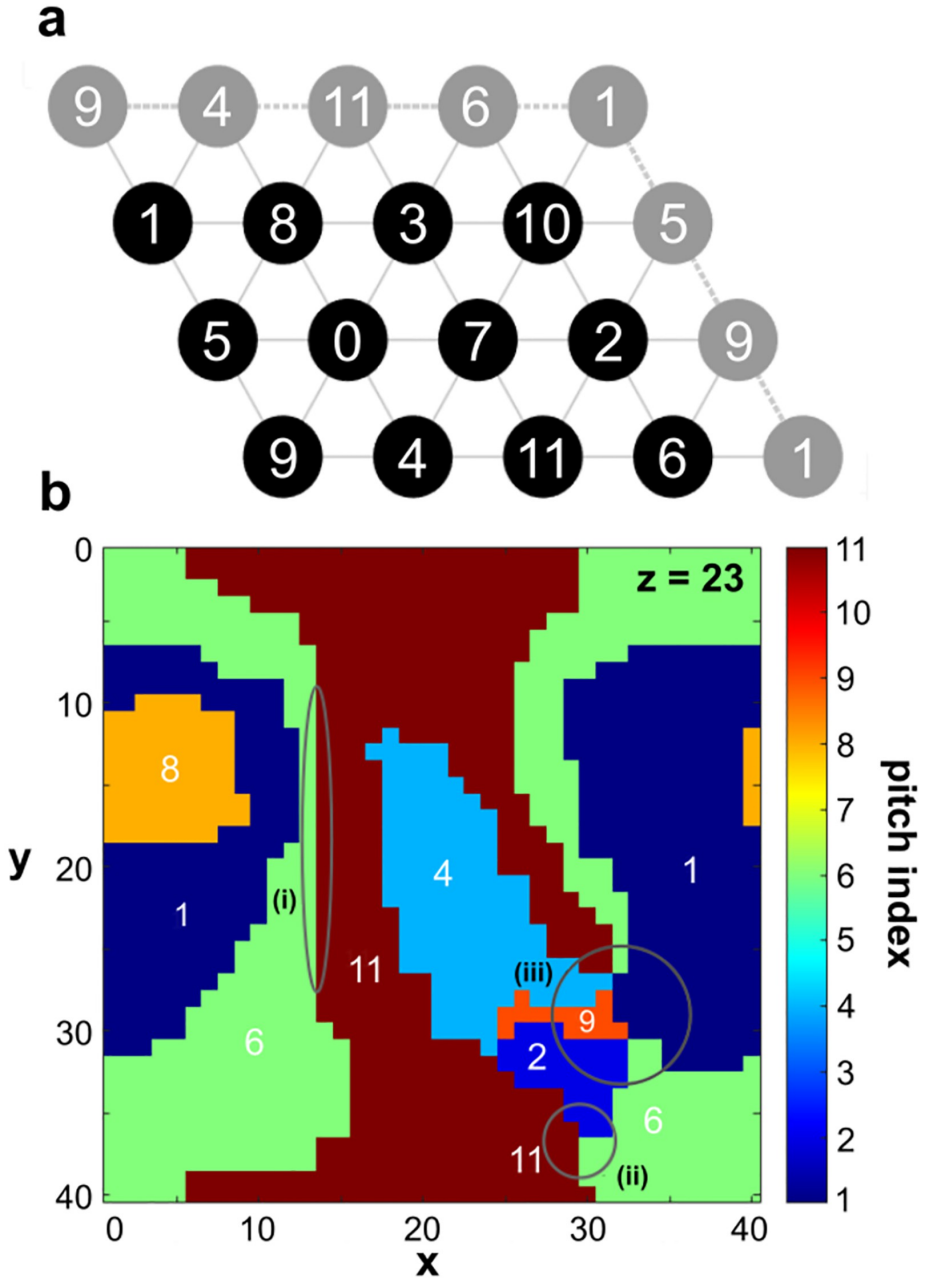
Arrangement of pitches in the quenched state. (a) The Tonnetz, with pitch indices labeling nodes and edges representing consonant intervals. Edges connecting nodes across the graph are indicated by repeated nodes shown in gray. (b) A 2D slice from the simulated 3D lattice at *T* = 5 *D*_0_ following a quench at *τ*_*Q*_ = 4368*t*_0_. Each pixel represents a lattice site, colored according to pitch. (i) shows a domain wall with an interval of p4 or p5, (ii) shows a minor triad vortex, (iii) encircles another triad vortex and two tetrad vortices.

Much of traditional Western harmony can be understood by reference to a graph known as the Tonnetz illustrating significant relationships between pitch classes, shown in Fig 6(a). In its earliest form, the Tonnetz was first described by Euler [19], and more recently is the basis of the Neo-Riemannian theory. This theory originated from the ideas of Hugo Riemann and Arthur von Oettingen in the 19th century, was largely established in the 20th century by music theorist David Lewin, and was further developed by Brian Hyer, Richard Cohn, Henry Klumpenhouwer and others [43]. In recent years, the structure of the Tonnetz and related graphs has been studied by Tymoczko and others [44–46]. We will see below that neighboring pitches on the Tonnetz are also predominantly the neighboring pitches on the quenched tone lattice, which yields a strong connection between our results and traditional harmony.

Each node on the Tonnetz (Fig 6(a)) is labelled by a pitch class, with edges connecting those nodes representing significant pitch differences (or “intervals”). Topologically, the Tonnetz is on a torus, with the nodes connected from top-to-bottom, and left-to-right, as shown by the gray nodes that repeat nodes from the other side.

The horizontal lines on the Tonnetz shown in Fig 6(a) connect nodes with pitch difference Δ*p* = ±7/12 mod 1 (or that connect pitch indices that differ by ±7 mod 12). As discussed above, these intervals are called a perfect fifth (p5) or a perfect fourth (p4), and occur near the second deepest minima of *D*(Δ*p*). Due to the boundary conditions at the edges of the Tonnetz, we can proceed 12 steps along the horizontal edges and return back to the initial node, a cycle referred to as the “circle of fifths.”

The edges in Fig 6(a) shown going up and to the right connect nodes with pitch difference Δ*p* = ±3/12 mod 1 (or pitch index difference ±3 mod 12). The edges in the other diagonal direction, going up and to the left, connect nodes with Δ*p* = ±4/12 mod 1. The intervals in the two diagonal directions are called a minor third (m3) and a major third (M3) respectively. These pitch differences are fairly close to minima of *D*(Δ*p*) arising from pitches with frequency in ratios 5/4 and 6/5 (log_2_(5/4) ≈ 0.322 ∼ 4/12 and log_2_(6/5) ≈ 0.263 ∼ 3/12.) Referring back to Fig 1, we can see that these intervals correspond to the next two deepest minima in *D*(Δ*p*). Thus the Tonnetz illustrates the three most consonant intervals [47]: p4, M3, and m3. And in the other direction, each edge represents the “inversion” of those intervals: p5, the minor sixth (m6), and major sixth (M6), respectively.

When multiple pitches are sounded simultaneously, they form a chord. The most consonant two-note chords, or “dyads” are p4, M3, and m3 and their inversions as discussed above, and shown on the Tonnetz (Fig 6(a)) [47]. A consonant three-note chord, or “triad,” can be constructed by choosing three pitches that each form consonant intervals with the other two. Starting with the consonant interval p5 (e.g. 0–7), we can add a third pitch at an interval M3 above the lower pitch (the “root”), and therefore a m3 below the higher pitch (e.g. 0–4-7). This chord is referred to as a major triad. Similarly, the third pitch can be placed a m3 above the root, yielding a minor triad (e.g. 0–3-7). Note that major (minor) triads appear as downward- (upward-) pointing triangles on the Tonnetz in Fig 6(a). Major and minor triads form the backbone of much of traditional Western harmony.

The major and minor triads provide a basis for understanding which pitches are sounded together; the next question is how those chords are arranged in time. In short, chords adjacent in time tend to be near each other on the Tonnetz. This concept is the foundation of Neo-Riemannian theory, in which harmonic progressions are described by transitions between triangles on the Tonnetz sharing one or two nodes.

Historically, common chord progressions have relied largely on moving from one triad to a neighboring triad to the left or right, either changing between major to minor (adjacent upwards and downwards triangles sharing two nodes), or remaining either major or minor (adjacent both upwards or both downwards triangles sharing one node) [48]. The seven pitches in three adjacent (side-by-side) upwards or downwards triangles form the common “diatonic scale” used in Western music. Chord progressions moving amongst the six major and minor triads formed by these seven pitches yield “tonal” music in a particular “key.” Alternatively, other triads can be included by translating farther left or right, or moving to a triad above or below on the Tonnetz. Such a jump could result in a “modulation” to a different key if subsequent chords remain near the new tonal center, or can result in music that is more atonal, that is not described by a single tonal center or key.

Now we will return to our simulation results, and discuss how structures emerge with similarity to those in the traditional theory of musical harmony. Results are shown at *T* = 1 − 5*D*_0_ following a quench, when the arrangement of pitches on the lattice has become largely stable (see S1, S3, and S5 Videos.)

First, we will see that neighboring pitch domains tend to be consonant intervals, and that vortices are analogous to chords. Fig 6(b) shows one 2D slice of the quenched state from a simulation run. Each pixel represents one site on the lattice, and is colored according to its pitch. At this low temperature, all the pitches in each domain appear very close to the same value, yielding regions of nearly uniform color. In this slice, there are seven distinct pitches labelled by pitch indices. Note that neighboring domains often differ by ±7 mod 12 (consonant intervals p5 or p4), as indicated in region (i). Intervals of ±3 mod 12 or ±4 mod 12 also appear, corresponding to the somewhat less consonant intervals m3, M3, m6 or M6. In the full quenched 3D lattice for this run, there were 25,020 such consonant neighbors. The more dissonant intervals with pitch index difference 1, 2, 6, 10, and 11 (called semitone or m2, whole tone or M2, tritone, m7 and M7, respectively) almost never appear. In this slice, there is one such dissonant pixel boundary. In the full 3D lattice, there were 385 such dissonant neighbors.

The points where three or more domains meet at a point in Fig 6(b) are topologically distinct vortex defects, which we can interpret as chords. Several examples appear in the circled regions (ii) and (iii). Region (ii) indicates a vortex where three pitch domains (11–2-6) meet. Starting from pitch 11, these three pitches form a minor triad, appearing as an upward pointing triangle on the Tonnetz in Fig 6(a). Recall that because the interaction function *D*(Δ*p*) contains multiple local minima, vortices will proceed by discrete jumps around the core, as opposed to continuous rotation. A vortex that proceeds around the core via the pitches of a major or minor triad is a particularly low-dissonance (or “low-energy”) configuration, as all the pitches are mutually consonant. The circled region (iii) also contains a triad, in this case, the major triad 9–1-4. Major and minor triads are the most common triads in the quenched state. The next most common triad is a “suspended” triad of the form 0–5-7. Though this triad contains the relatively dissonant and uncommon whole tone, the whole tone boundary tends to be very short, and bounded on each end by a suspended triad or similar chord. For example, the single whole tone boundary in Fig 6(b) is part of a suspended triad with root of 9. This chord forms a continuous, though not closed, path on the Tonnetz. The other possible triad that can form a closed path on the Tonnetz is the augmented triad 0–4-8. These do occur in the quenched state, though only rarely because p4 and p5 are strongly favored over the M3 intervals forming the augmented triad. In this full 3D lattice, 90% of triads form continuous paths on the Tonnetz, and 49% form closed paths on the Tonnetz.

Note that because of the cubic lattice, there can be at most four pitches around a point. In principle though, a vortex can form with any number of pitches surrounding a point. When such a vortex forms in this simulation, it must appear as multiple closely spaced vortices with three or four pitch classes.

Vortices where four pitch domains meet can be interpreted as a four-note chord, or tetrad. It should be noted, however, that in this case each pitch only interacts with two of the three others. Therefore, this defect could instead be viewed as four overlapping dyads. It is not possible for a tetrad with four distinct pitch classes to be composed of consonant intervals (m3, M3, p4, and their inversions) between all pairs of pitches. In a four-fold vortex, each pitch tends to be consonant with its neighbors, but not with the fourth pitch that it does not border. There are two such examples in region (iii): 1–6-11-4 and 1–9-2-6. The tetrads that appear in the simulation results can take on different forms, but, like the triads, they often form a closed path on the Tonnetz. This closed path can be in the shape of a parallelogram covering two adjacent triangles (as in 1–9-2-6), or along a path that circulates around the boundaries of the Tonnetz (as in 4–11-6-1). In this full 3D lattice, 99% of tetrads form continuous paths on the Tonnetz, and 59% form closed paths on the Tonnetz.

In traditional Western music, the most common tetrads with four distinct pitch classes are the so-called “seventh” chords which add a M7 or m7 (pitch index difference 10 or 11 mod 12) above the lowest pitch of a standard major or minor triad [48]. Of the three orientations of parallelograms on the Tonnetz, we expect the two orientations with two horizontal edges (p5/p4) to occur much more commonly than the orientation with all diagonal edges (m3/M3). These two parallelograms each represent a seventh chord: a m7 above a minor triad (the minor seventh chord), or a M7 above a major triad (the major seventh chord). Notably, however, the commonly used major-minor, or “dominant” seventh chord, with a m7 above a major triad, appears very rarely in our simulation results.

The most common way for a vortex tetrad to form a closed path around the boundaries of the Tonnetz is for the four pitches to be arranged along a horizontal line on the Tonnetz, with the loop closed by a diagonal connection, so there are three p4 or p5 intervals, and one m3 or M3 among the four pitches. This is the case in the example above (4–11-6-1). If we instead view the four-pitch defect as a set of four dyads, we could view such an arrangement as forming the backbone for a tonal harmony by including the root and p5 of the three most important triads in a given key. These are the triad based on the tonal center itself (the tonic, or I chord), and the triads a p4 and p5 above the tonic (the sub-dominant or IV chord, and the dominant or V chord, respectively). Alternatively, if all four pitches are sounded together, this combination of pitches is referred to as a suspended seventh chord, consisting of a root pitch, and a p4, p5, and m7 above the root. Another possible tetrad forming a closed path on the Tonnetz is the diminished seventh chord 0–3-6-9. This tetrad rarely occurs in the quenched state again because these m3 intervals are not favored as compared to p4 and p5.

Because of the mapping between the arrangement of pitch domains in the simulation and the structure of the Tonnetz, it should not be surprising that compact regions within the simulated lattice form commonly used scales, such as the 7-note diatonic scale (e.g. C D E F G A B, or in pitch indices, 0 2 4 5 7 9 11). For example, the slice shown in Fig 6(b) happens to contain exactly the seven notes of a major diatonic scale starting from pitch index 9. Not every 2D slice has this property, but it is generally true that these scales can be found in connected, compact regions of the lattice.

**Fig 7:**
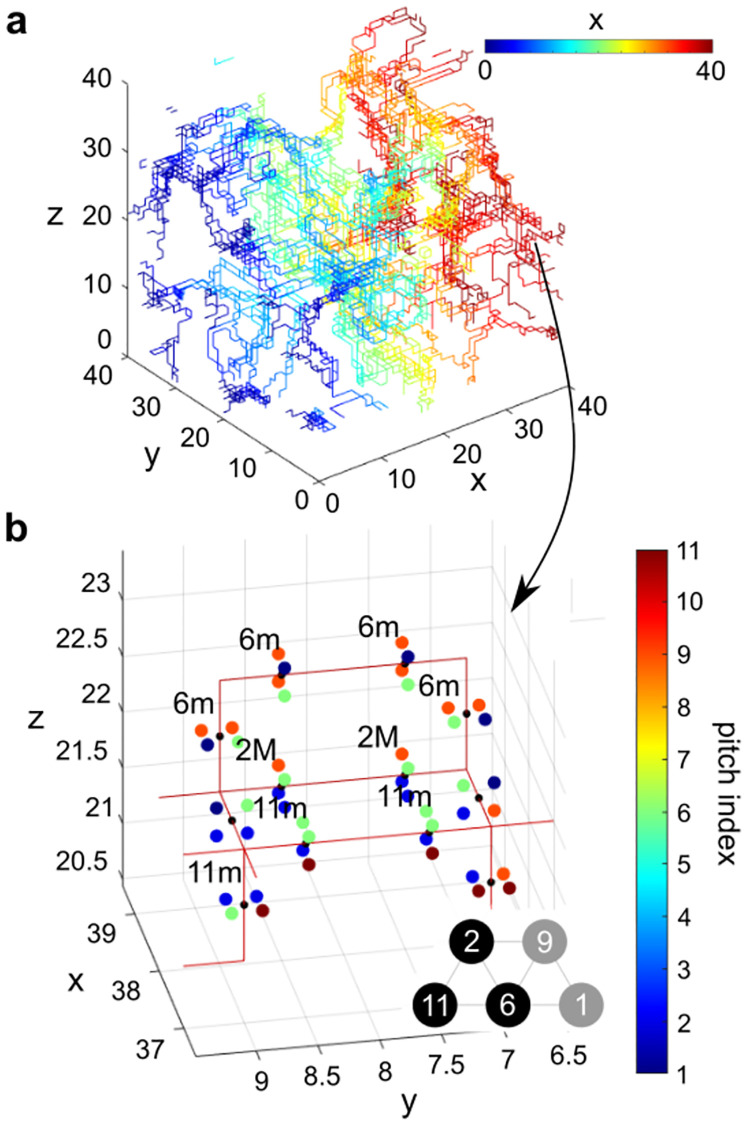
Vortex strings in the quenched state. (a) Network of vortex strings in the quenched state of a simulation run with *τ*_*Q*_ = 1638*t*_0_. Color gradient is applied along the x-axis to improve clarity. (b) Zoom in of a small region of (a), showing vortex strings in red. Pitches surrounding the vortex at each string segment are indicated by four colored dots around the string segment. Vortices corresponding to major or minor triads are labeled by the root pitch index and M or m respectively. Inset shows a portion of the Tonnetz spanning the pitches appearing in (b).

Moving from the 2D slice to the full 3D lattice, the vortex triads and tetrads become vortex strings tracing out a network through the simulated volume. Now, instead of finding vortices at the corners of pixels in 2D, we identify vortices at the edges of 3D voxels, where each lattice site is a small cube. Four voxels can share an edge along one of the three Cartesian axes. If there are three or more distinct pitch class regions surrounding a shared edge, we identify that edge as a segment of a vortex string. Fig 7(a) plots all such vortex string segments. A color gradient is applied along the x-axis to improve clarity. String-like branching paths are visible threading through the volume. Bundles of nearby strings indicate closely spaced vortices that cannot merge due to the limitations of the cubic lattice. Recall that periodic boundary conditions are imposed on the simulation volume, so strings that end at one face continue at the opposite face. Videos of the formation of the vortex strings in three quenches with different *τ*_*Q*_ are shown in S2, S4 and S6 Videos. By the end of the quench, it can be seen that the structure of the vortex strings is fairly stable, with only small fluctuations persisting.

Due to topology, the vortex strings cannot end, but can form branches into other triad or tetrad strings. For example, a vortex string forms along the line where three 3D pitch domains meet. If a fourth pitch domain comes in to meet along that line, then new strings will branch out where three or four pitch domains come together. Because (in this example) all of the chords meeting at this branching point are composed from some subset of the same four pitches, they all must share at least two pitches. Therefore, all of these chords are adjacent on the Tonnetz. To quantify this, we extract statistics from the junctions between chords in Fig 7(a). At 95% of these junctions, the chords on either side conserve two or three pitches, and at 90% of junctions, the pitches added to either chord are adjacent to the conserved pitches on the Tonnetz.

According to neo-Riemannian music theory, the chord progressions often found in music can be described by transitions between neighboring chords on the Tonnetz. These neighboring chords share at least one pitch, and the pitches unique to each chord are adjacent to the conserved pitches on the Tonnetz. Therefore, the transformations between chords in neo-Riemannian theory proceed according to a similar pattern as the junctions between vortex strings in the simulation here. As such, a path along the branching vortex strings can be considered to be a chord progression. To illustrate this, we zoom in on a small volume of the simulation results in Fig 7(b). The string segments in this volume are shown in red. The midpoint of each segment is indicated by a black dot, and the pitches of the four surrounding voxels are indicated by the color of the four larger dots. When the vortex corresponds to a major or minor triad, it is labeled by the root pitch index followed by M or m, respectively. Note the voxels themselves fill an entire cube between integer values on the axes—the dots indicating the pitches of the voxels are not shown at the center of the voxel but are shifted closer to the string segment for clarity.

Based on the arrangement of pitches shown in Fig 7(b) we can form a rough picture of the pitch domains that meet here. The prevalence of red dots at the back of the figure (*x* > 38) indicates that there is a domain with pitch index 9 in that region. At *x* < 38 and *z* > 22 we see a grouping of green dots, indicating a domain with pitch index 6.

Four string junctions can be found in Fig 7(b). As an example, we focus on the junction at (*x*, *y*, *z*) = (38, 7, 22) on the right side of the figure, where the pitches on all branches are visible in the figure. Two of the vortex strings meeting at that point correspond to triads 6m (6-9-1) and 2M (2-6-9). These two chords share two pitches, and are found neighboring side-to-side on the Tonnetz. The third vortex string meeting at this point is a tetrad (2-9-6-1) which can be interpreted as a major seventh chord with a root of pitch index 2 (2M^7^). As these chords all share two or three pitch classes, a transition between any two chords would constitute a natural chord progression.

### Conclusions

We have investigated a new way of using the tools of statistical mechanics to understand the structure of musical harmony. By constructing a fundamental model of musical harmony that resembles the well-studied XY model, we have seen how the expected emergent phenomena provide new insight into music theory. We observe a phase transition from disordered sound to a discrete set of pitches, with critical behavior consistent with the universal exponents for this type of system. Quenching through the transition at a finite rate precludes reaching the single-pitch ground state, and instead results in a frozen-in state threaded by vortex strings. This quenched state repeatably displays domains of twelve equally spaced pitch classes per octave, as is the case in Western music. Furthermore, the size of these pitch domains and concentration of vortex strings follows the behavior predicted by the KZM. We see that the emergent structure of the vortices and vortex strings is similar to harmonic structures and progressions found in Western musical tradition.

The similarity between the arrangement of pitches in the quenched state and in traditional Western harmony suggests a connection between the two, though we have not yet discussed the reasons why the musical systems arising in human cultures should correspond to the quenched state of this model. Temperature, defined here as setting the trade-off between reduced dissonance and increased compositional possibility, is presumably culturally determined. It seems unlikely, however, that musical systems are defined by a rapid quench of this temperature. Instead, we suggest that the freezing-in of a higher entropy state represents the practical need to concretely codify a system of music. The correlations seen in the non-equilibrium quenched state reflect those seen in equilibrium at some higher temperature. But at that higher temperature, the state of the system is dynamic, with vortices and anti-vortices forming and annihilating. Such a dynamic state does not lend itself well to the practice of music. Instead, the choice of one particular static configuration represents the establishment of a musical system that will be common to many musicians, to which instruments can be built to accommodate, and that allows consistent pedagogy for students. Indeed, it is natural to see this freezing-in process as an outcome of human culture: once a static configuration is chosen, its widespread use will make it difficult to change.

The differences between the emergent structures in this model and established music theory will provide interesting opportunities to extend or generalize our understanding of musical structure. Viewing the vortex strings in the quenched state as possible chord progressions, composers may choose pitches from sites along a path traversing the strings to create music. These chord progressions make a connection with traditional triadic harmonies of Western music, but also emphasize other closed loops on the Tonnetz that offer new concepts of harmony. The dissonance function *D*(Δ*p*) used here was based on a reasonable choice of parameters, which led to the observed 12-fold octave division. The results of Ref. [1], however, indicate that other parameters specifying *D*(Δ*p*) will result in other ordered phases, such as with 5, 7, or 31 pitches per octave. These octave divisions are used in other traditional or experimental musical systems, and thus these results indicate a pathway to exploring harmony in those systems as well. We have seen that the universality of critical behavior allows us to link realms as seemingly unrelated as music theory and cosmology, which may provide both novel technical approaches and artistic inspiration for the composition, performance, and appreciation of music.

## Methods

### Calculation of dissonance function

There are several approaches to quantifying the human perceptions of consonance and dissonance, with controversy around numerous subtleties and disagreement even about the definitions of the terms. However, the approach we follow here, based on the ideas of Helmholtz [28], and later the work of Plomp and Levelt [29], and Sethares [30], yields a closed form dissonance function *D*(Δ*p*) that captures the essential features of the results of most other approaches.

We follow closely the method used in Ref. [1] with a parametrized value of the critical band width *w*_*c*_. The perceived dissonance of two pure tones with frequency *f*_1_ and *f*_2_ separated by interval Δ*p* = log_2_(*f*_2_/*f*_1_) was measured experimentally by Plomp and Levelt. We capture the trends of their measured curves with an appropriately shaped function $d\left(f_{1},f_{2}\right)=e^{-{\left[\text{ln}\left(|\Delta p|/w_{c}\right)\right]}^{2}}/w_{c}$. The maximum of this curve is at *w*_*c*_ which specifies the so-called critical band width. To match the experimentally measured dependence on the lower of the two pitches, we parameterize *w*_*c*_ = 5.67min(*f*_1_, *f*_2_)^−0.68^ with *f* in Hz.

We then sum over the pairs of partials of two sawtooth waveforms with amplitudes *α*_*n*_ = 1/*n*, and frequencies *nf*_1_ and *nf*_2_ (*n* = 1, 2, 3, …, 10). This yields the dissonance function

$$D_{0}\left(\Delta p\right)=\sum_{n,m}l_{n,m}d\left(nf_{1},mf_{2}\right),$$

where *l*_*n*,*m*_ = min(*α*_*n*_, *α*_*m*_)^0.606^ is the perceived loudness as estimated by Sethares [30]. Though in principle due the frequency dependence of *w*_*c*_, the detailed shape of this function would depend on the lesser of *f*_1_ and *f*_2_, we fix that root frequency to be a typical value of 424 Hz. Finally, we impose octave-wise periodicity by summing each octave of *D*_0_:

$$D\left(\Delta p\right)=\sum_{n=-\infty }^{\infty }D_{0}\left(\Delta p+n\right).$$

### Simulation method

The simulation method is similar to that used in Ref. [1], with the description below adapted from that work. We place the tones on a 40 × 40 × 40 three-dimensional cubic lattice with periodic boundary conditions at the edges, and with interactions only with the 6 nearest neighbors.

Via the analogy between dissonance and energy, the gradient of dissonance can be viewed as a force, pulling intervals towards lower dissonance. Assigning the tones a mass *m* and damping constant *ζ* we can write a stochastic equation of motion

$$m\ddot{p_{i}}+\zeta \dot{p_{i}}+dD_{i}/dp=\sqrt{2T\zeta }\eta_{i},$$

where *D*_*i*_ is the total dissonance of tone *i* with its neighbors, and *η*_*i*_ is a Gaussian white noise scalar with variance 1. Evolving this system of equations for all *i* yields the dynamics of the system at temperature *T*. We choose $m=4\;D_{0}t_{0}^{2}$, which yields a typical oscillation frequency *ω* ∼ 400 1/*t*_0_, or about 60 periods within time *t*_0_, for a pitch near a minimum of *D*(Δ*p*) with respect to its neighbors. We set *ζ* = *mω* ≈ 1600 *D*_0_*t*_0_ such that the dynamics are near critical damping, so that the system rapidly thermalizes.

Simulating the Langevin dynamics of the system explores the phase space around one local minimum of the total dissonance. In order to fully sample the phase space, we also include Monte Carlo (MC) steps, chosen via the Metropolis algorithm, resulting in larger jumps in phase space. Here, a lattice site *i* is chosen at random, and we calculate the change in total dissonance *δD* if the pitch *p*_*i*_ were changed to a new, randomly selected value *p*_*i*_ + Δ*p*, where Δ*p* is drawn from the normalized probability distribution proportional to exp(−*D*(Δ*p*)/(5*D*_0_)). If exp(−*δD*/*T*) < *r*, with 0 ≤ *r* < 1 a uniformly distributed random number, then the change is rejected; otherwise the change is accepted. Further illustration and characterization of the simulation methods are given in S1 Text.

At high temperature, the set of tones is initialized into a disordered state with pitches *p*_*i*_ drawn from a uniform distribution. The simulation proceeds by performing a number (in this case, 10,000) of MC steps followed by thermalization via Langevin dynamics at a fixed temperature *T*. This sequence, which we define to take one unit of time *t*_0_, is then repeated typically *n*_*T*_ = 10 − 100 times at each temperature. Once *n*_*T*_ repetitions are completed at a fixed *T*, the temperature is changed by a temperature step Δ*T* (typically Δ*T* = (0.01 − .5)*D*_0_), and another *n*_*T*_ simulation steps are performed. The values of Δ*T* and *n*_*T*_ therefore set the inverse quench rate *τ*_*Q*_ = *n*_*T*_*T*_*c*_*t*_0_/Δ*T*.

## Supporting information

S1 Text Supplemental information. Section 1 presents examples and characterization of the simulation methods. Section 2 presents further characterization of the phase transition. Section 3 presents additional results to demonstrate the repeatability and generality of the results.
(PDF)

S1 Video Slow quench. Video of a quench at *τ*_*Q*_ = 13650 *t*_0_. A histogram of all pitches and the lattice itself are plotted vs. temperature with the same color scale as Fig 2.
(MP4)

S2 Video Vortex strings during a slow quench. Video of the vortex strings during a quench at *τ*_*Q*_ = 13650 *t*_0_. The vortex strings are plotted as in Fig 7, starting from a temperature slightly below *T*_*c*_.
(MP4)

S3 Video Medium quench. Video of a quench at *τ*_*Q*_ = 1638 *t*_0_. A histogram of all pitches and the lattice itself are plotted vs. temperature with the same color scale as Fig 2. The simulation continues for some additional time after reaching *T* = 1 *D*_0_ to show the stability of the pitch domains in the quenched state.
(MP4)

S4 Video Vortex strings during a medium quench. Video of the vortex strings during a quench at *τ*_*Q*_ = 1638 *t*_0_. The vortex strings are plotted as in Fig 7, starting from a temperature slightly below *T*_*c*_. The simulation continues for some additional time after reaching *T* = 1 *D*_0_ to show the stability of the vortex strings in the quenched state.
(MP4)

S5 Video Fast quench. Video of a quench at *τ*_*Q*_ = 546 *t*_0_. A histogram of all pitches and the lattice itself are plotted vs. temperature with the same color scale as Fig 2.
(MP4)

S6 Video Vortex strings during a fast quench. Video of the vortex strings during a quench at *τ*_*Q*_ = 546 *t*_0_. The vortex strings are plotted as in Fig 7, starting from a temperature slightly below *T*_*c*_.
(MP4)

S7 Video Simulation example: Relaxation of a small perturbation at *T* < *T*_*c*_ using Langevin dynamics only. Left plot shows the interior of the simulation volume with a restricted color scale to show detail. Right plot shows a histogram of all pitches in the lattice.
(MP4)

S8 Video Simulation example: Relaxation of a small perturbation at *T* < *T*_*c*_ using MC steps only. Left plot shows the interior of the simulation volume with a restricted color scale to show detail (the color of sites with *p* ≈ 1/12 and 11/12 are oversaturated at the color scale limits). Right plot shows a histogram of all pitches in the lattice.
(MP4)

S9 Video Simulation example: Relaxation of a small perturbation at *T* < *T*_*c*_ using both MC steps and Langevin dynamics. Left plot shows the interior of the simulation volume with a restricted color scale to show detail (the color of sites with *p* ≈ 1/12 and 11/12 are oversaturated at the color scale limits). Right plot shows a histogram of all pitches in the lattice.
(MP4)

S10 Video Simulation example: Relaxation of a perturbation away from the disordered state at *T* > *T*_*c*_ using both MC steps and Langevin dynamics. Left plot shows the interior of the simulation volume. Right plot shows a histogram of all pitches in the lattice.
(MP4)

S11 Video Simulation example: Relaxation of a perturbation away from the disordered state at *T* > *T*_*c*_ using Langevin dynamics only. Left plot shows the interior of the simulation volume. Right plot shows a histogram of all pitches in the lattice.
(MP4)

S12 Video Simulation example: Relaxation of a perturbation away from the disordered state at *T* > *T*_*c*_ using MC steps only. Left plot shows the interior of the simulation volume. Right plot shows a histogram of all pitches in the lattice.
(MP4)

S13 Video Very slow quench. Video of a quench at *τ*_*Q*_ = 273000 *t*_0_. A histogram of all pitches and the lattice itself are plotted vs. temperature with the same color scale as Fig 2.
(MP4)

## References

[1] Berezovsky J. The structure of musical harmony as an ordered phase of sound: A statistical mechanics approach to music theory. Science Advances. 2019;5(5). doi: 10.1126/sciadv.aav8490 31114802 PMC6524979

[2] Kibble TWB. Topology of cosmic domains and strings. Journal of Physics A: Mathematical and General. 1976;9(8):1387–1398. doi: 10.1088/0305-4470/9/8/029

[3] del Campo A, Retzker A, Plenio MB. The inhomogenous Kibble-Zurek mechanism: vortex nucleation during Bose-Einstein condensation. New Journal of Physics. 2011;13(083022).

[4] Hendry PC, Lawson NS, Lee RAM, McClintock PVE, Williams CDH. Generation of defects in superfluid 4He as an analogue of the formation of cosmic strings. Nature. 1994;368:047101. doi: 10.1038/368315a0

[5] Alden JS, Tsen AW, Huang PY, Hovden R, Brown L, Park J, et al. Strain solitons and topological defects in bilayer graphene. Proceedings of the National Academy of Sciences of the United States of America. 2013;110(28):11256–11260. doi: 10.1073/pnas.1309394110 23798395 PMC3710814

[6] Griffin SM, Lilienblum M, Delaney KT, Kumagai Y, Fiebig M, Spaldin NA. Scaling Behavior and Beyond Equilibrium in the Hexagonal Manganites. Phys Rev X. 2012;2:041022. doi: 10.1103/PhysRevX.2.041022

[7] Casado S, González-Viñas W, Mancini H. Testing the Kibble-Zurek mechanism in Rayleigh-Bénard convection. Phys Rev E. 2006;74:047101. doi: 10.1103/PhysRevE.74.047101 17155216

[8] Das A, Sabbatini J, Zurek WH. Winding up superfluid in a torus via Bose Einstein condensation. Scientific reports. 2012;2. doi: 10.1038/srep00352 PMC3324982 22500209

[9] Bäuerle C, Bunkov YM, Fisher S, Godfrin H, Pickett G. Laboratory simulation of cosmic string formation in the early Universe using superfluid 3He. Nature. 1996;382(6589):332–334. doi: 10.1038/382332a0

[10] Ruutu V, Eltsov V, Gill A, Kibble T, Krusius M, Makhlin YG, et al. Vortex formation in neutron-irradiated superfluid 3He as an analogue of cosmological defect formation. Nature. 1996;382(6589):334–336. doi: 10.1038/382334a0

[11] Monaco R, Mygind J, Rivers R, Koshelets V. Spontaneous fluxoid formation in superconducting loops. Physical Review B. 2009;80(18):180501. doi: 10.1103/PhysRevB.80.180501

[12] Weiler CN, Neely TW, Scherer DR, Bradley AS, Davis MJ, Anderson BP. Spontaneous vortices in the formation of Bose–Einstein condensates. Nature. 2008;455. doi: 10.1038/nature07334

[13] Maniv A, Polturak E, Koren G. Observation of magnetic flux generated spontaneously during a rapid quench of superconducting films. Physical review letters. 2003;91(19):197001. doi: 10.1103/PhysRevLett.91.197001 14611602

[14] Chuang I, Durrer R, Turok N, Yurke B. Cosmology in the laboratory: Defect dynamics in liquid crystals. Science. 1991;251(4999):1336–1342. doi: 10.1126/science.251.4999.1336 17816188

[15] Ducci S, Ramazza PL, González-Viñas W, Arecchi F. Order parameter fragmentation after a symmetry-breaking transition. Physical Review Letters. 1999;83(25):5210. doi: 10.1103/PhysRevLett.83.5210

[16] Vilenkin A, Everett AE. Cosmic strings and domain walls in models with Goldstone and pseudo-Goldstone bosons. Physical Review Letters. 1982;48(26):1867. doi: 10.1103/PhysRevLett.48.1867

[17] Sikivie P. Axions, Domain Walls, and the Early Universe. Phys Rev Lett. 1982;48:1156–1159. doi: 10.1103/PhysRevLett.48.1156

[18] Blasone M. A Physicist’s view on Chopin’s Études. The European Physical Journal Special Topics. 2017;226(12):2715–2728. doi: 10.1140/epjst/e2017-70008-6

[19] Euler L. Tentamen Novae Theoriae Musicae: Ex Certissimus Harmoniae Principiis Dilucide Expositae. Ex typographia Academiae scientiarum; 1739.

[20] Fokker AD. “Unison Vectors and Periodicity Blocks in 3-Dimensional (3-5-7-) Harmonic Lattice of Notes”. Proceedings of the Koninklijke Nederlandse Akademie van Wetenschappen Series B—Physical Sciences. 1969;72(3):153.

[21] Pires A, Gouvea M. Phase transition and spin dynamics in the two-dimensional easy-plane ferromagnet. Physical Review B. 1993;48(17):12698. doi: 10.1103/PhysRevB.48.12698 10007640

[22] Zurek W. Cosmological experiments in superfluid helium. Nature. 1985;317:505–509. doi: 10.1038/317505a0

[23] McDermott JH, Schultz AF, Undurraga EA, Godoy RA. “Indifference to Dissonance in Native Amazonians Reveals Cultural Variation in Music Perception”. Nature. 2016;535(7613):547–550. doi: 10.1038/nature18635 27409816

[24] Proverbio AM, Orlandi A, Pisanu F. “Brain processing of consonance/dissonance in musicians and controls: a hemispheric asymmetry revisited”. European Journal of Neuroscience. 2016;44(6):2340–2356. doi: 10.1111/ejn.13330 27421883

[25] Meyer LB. Emotion and meaning in music. University of Chicago Press; 2008.

[26] Berg RE, Stork DG. The Physics of Sound. Pearson Education India; 1990.

[27] Tramo MJ, Cariani PA, Koh CK, Makris N, Braida LD. Neurophysiology and neuroanatomy of pitch perception: auditory cortex. Annals of the New York Academy of Sciences. 2005;1060(1):148–174. doi: 10.1196/annals.1360.011 16597761

[28] Helmholtz H. On the Sensations of Tone. Dover Books on Music. Dover Publications; 2013. Available from: https://books.google.com/books?id=QNfCAgAAQBAJ.

[29] Plomp R, Levelt W. Tonal consonance and critical bandwidth. The Journal of the Acoustical Society of America. 1965;38 4:548–60. doi: 10.1121/1.1909741 5831012

[30] Sethares W. Local consonance and the relationship between timbre and scale. The Journal of the Acoustical Society of America. 1993;94. doi: 10.1121/1.408175

[31] Steinhardt PJ, Nelson DR, Ronchetti M. Bond-orientational order in liquids and glasses. Physical Review B. 1983;28(2):784. doi: 10.1103/PhysRevB.28.784

[32] Deutschländer S, Dillmann P, Maret G, Keim P. Kibble–Zurek mechanism in colloidal monolayers. Proceedings of the National Academy of Sciences. 2015;112(22):6925–6930. doi: 10.1073/pnas.1500763112 25902492 PMC4460445

[33] Miranda MA, et al. The Kibble-Zurek mechanism in a subcritical bifurcation. Phys: Condens Matter. 2013;25(40). doi: 10.1088/0953-8984/25/40/404208 24025325

[34] Dave SS, Srivastava AM. Formation of topological vortices during superfluid transition in a rotating vessel. A Letters Journal Exploring the Frontiers of Physics. 2019;126(31001). doi: 10.1209/0295-5075/126/31001

[35] Dodd ME, Hendry PC, Lawson NS, McClintock PVE, Williams CDH. Nonappearance of Vortices in Fast Mechanical Expansions of Liquid ^4^He through the Lambda Transition. Phys Rev Lett. 1998;81:3703–3706. doi: 10.1103/PhysRevLett.81.3703

[36] Miranda MA, Burguete J, Mancini H, González-Viñas W. Frozen dynamics and synchronization through a secondary symmetry-breaking bifurcation. Physical Review E. 2013;87(3):032902. doi: 10.1103/PhysRevE.87.032902

[37] Wu FY. The Potts model. Rev Mod Phys. 1982;54:235–268. doi: 10.1103/RevModPhys.54.235

[38] Herrmann H. Monte Carlo simulation of the three-dimensional Potts model. Zeitschrift für Physik B Condensed Matter. 1979;35(2):171–175.

[39] Gottlob AP, Hasenbusch M. Critical behaviour of the 3D XY-model: a Monte Carlo study. Physica A: Statistical Mechanics and its Applications. 1993;201(4):593–613. doi: 10.1016/0378-4371(93)90131-M

[40] Fisher ME, Barber MN. Scaling Theory for Finite-Size Effects in the Critical Region. Phys Rev Lett. 1972;28:1516–1519. doi: 10.1103/PhysRevLett.28.1516

[41] Privman V. Finite size scaling and numerical simulation of statistical systems. World Scientific; 1990.

[42] Campostrini M, Hasenbusch M, Pelissetto A, Rossi P, Vicari E. Critical behavior of the three-dimensional XY universality class. Physical Review B. 2001;63(21):214503. doi: 10.1103/PhysRevB.63.214503

[43] Cohn R. Introduction to Neo-Riemannian Theory: A Survey and a Historical Perspective. Journal of Music Theory. 1998;42(2):167–180. doi: 10.2307/843871

[44] Tymoczko D. The Generalized Tonnetz. Journal of Music Theory. 2012;56(1):1–52. doi: 10.1215/00222909-1546958

[45] Gollin E. Some aspects of three-dimensional ‘Tonnetze’. Journal of Music Theory. 1998; p. 195–206. doi: 10.2307/843873

[46] Callender C, Quinn I, Tymoczko D. Generalized Voice-Leading Spaces. Science. 2008;320(5874):346–348. doi: 10.1126/science.1153021 18420928

[47] Huron D. Interval-class content in equally tempered pitch-class sets: Common scales exhibit optimum tonal consonance. Music Perception. 1994;11(3):289–305. doi: 10.2307/40285624

[48] Moss FC. Transitions of tonality: a model-based corpus study. EPFL; 2019.

