# Supplementary material for: Critical behavior and the Kibble-Zurek mechanism in a musical phase transition
Source: PLoS One. 2023 Jan 23;18(1):e0280227. doi: 10.1371/journal.pone.0280227 (PMC9870160; doi:10.1371/journal.pone.0280227)
Supplement: S1 Text — Section 1 presents examples and characterization of the simulation methods. Section 2 presents further characterization of the phase transition. Section 3 presents additional results to demonstrate the repeatability and generality of the results. (PDF) [file pone.0280227.s001.pdf]

S1 Text: Supporting Text for “Critical behavior and the Kibble-Zurek mechanism in a musical phase transition”

December 21, 2022

1 Validation of simulation methods

Here we illustrate the simulation results with several examples and characterize the behavior of the simulation results.

1.1 Thermalization below T_c

We initialize the system with a small perturbation away from the ground state, and observe how the system thermalizes at temperatures below T_c. The functional form of the perturbation is chosen to yield a clear visualization of the relaxation, and since it should overlap with many normal modes of the system, should give a general picture of the thermalization process. We show the simulation as used in the main text, with both Monte Carlo (MC) steps and Langevin dynamics, and compare to MC steps only, and Langevin dynamics only. We will see that either method alone is very inefficient.

The $40 \times 40 \times 40$ lattice of pitches is initialized to

$$p(x, y, z) = 0.5 + 0.05 \exp\left(-\frac{(x-20)^2 + (y-20)^2 + (z-20)^2}{200}\right). \quad (1)$$

This function is close the ground state with uniform $p = 0.5$, but with a Gaussian perturbation centered in the middle of the cube. The amplitude of this perturbation is comparable to the width of the first minimum of $D(\Delta p)$ at $\Delta p = 0$. Certainly all nearest neighbors will have Δp well within that first potential well.

We first run the simulation using Langevin dynamics only (the MC steps are commented out), at temperature $T = 20D_0$. Figure 1 illustrates the simulation state at several times (see also S7 video). The left column plots half of the lattice sites with a color scale restricted to better show the details. Only half of the lattice is shown to make visible the perturbation in the interior. The right column shows a histogram of the values of p in the entire lattice. At $t = 0.002t_0$, the simulation has just begun, and the initial distribution as given

above is shown. By $t = 0.08t_0$, we see that the damped dynamics have begun to relax the perturbation back towards a uniform pitch, where all $\Delta p \approx 0$. At this time point, the histogram has mostly lost the artificially imposed asymmetry of the initial state. Finally, by $t = 0.2t_0$, the initial perturbation has totally thermalized to a quasi-equilibrium state where the pitch is fluctuating within the deepest minimum of $D(\Delta p)$ near $\Delta p = 0$.

As we will see below, however, the state seen at the bottom of Fig. 1 is not the actual equilibrium of the system, as excitation to other minima of $D(\Delta p)$ should also occur. The Langevin dynamics are an extremely inefficient way for this to occur because of the large potential barrier to escape from the lowest minimum. Musically, the barrier is meaningless: pitches to not have to be adjusted continuously. Composers and musicians can change a pitch from any value to any other value discontinuously. This illustrates why the MC steps are both necessary and physically justified.

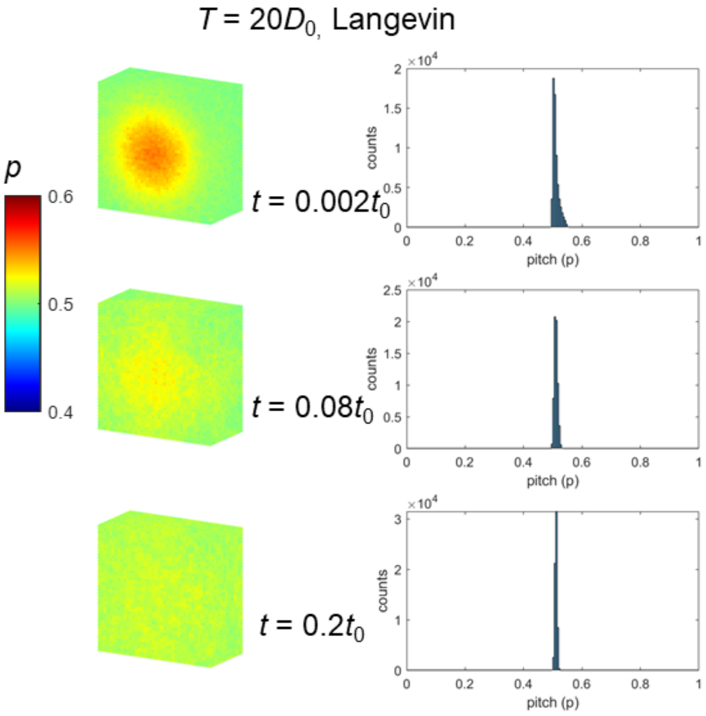

Figure 1: Simulation example: relaxation of a small perturbation at $T < T_c$ using Langevin dynamics only. Three time points are shown. Left column shows the interior of the simulation volume with a restricted color scale to show detail. Right column shows histograms of all pitches in the lattice.

Next, we run the same simulation as above, but with MC steps only (the code for the Langevin dynamics is commented out). Again, three snapshots are shown

in Fig. 2 (see also S8 video). Note that the total timescale here is much larger than in Fig. 1. At $t = t_0$, we see that the initial state is virtually unchanged, with just several lattice sites having jumped to other values. Compare to the Langevin dynamics above, where the initial perturbation had completely relaxed by this time $t = t_0$. As time progresses to $t = 50t_0$ and $t = 400t_0$, more sites have been excited to the next lowest minima near $\Delta p \approx \pm 5/12$. We will show below that by $t = 400t_0$ these excitations have largely equilibrated. However, the initial perturbation is only slightly relaxed by $t = 400t_0$. The asymmetry present in the original histogram peak is still present, and is now replicated in the side peaks. So we see that the MC steps are very inefficient at relaxing these small gradual perturbations.

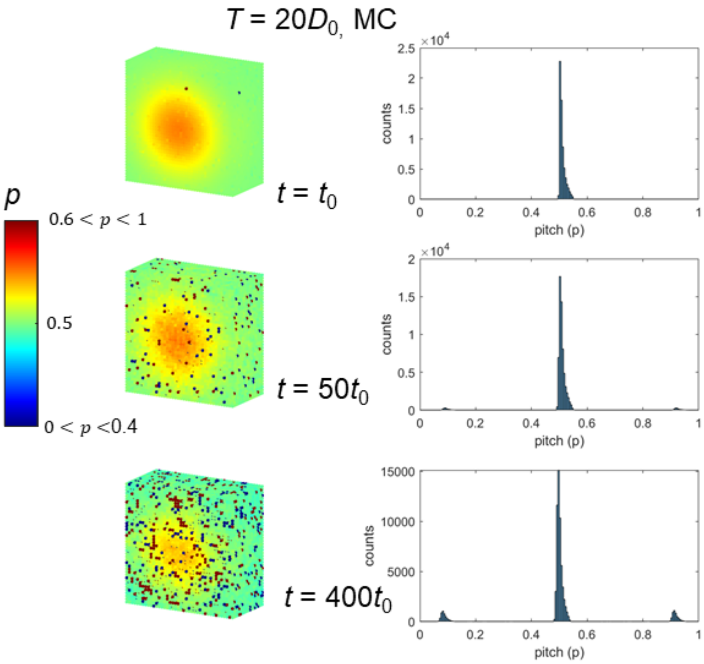

Figure 2: Simulation example: relaxation of a small perturbation at $T < T_c$ using MC steps only. Three time points are shown. Left column shows the interior of the simulation volume with a restricted color scale to show detail (the color of sites with $p \approx 1/12$ and $11/12$ are oversaturated at the color scale limits). Right column shows histograms of all pitches in the lattice.

The separate shortcomings of the MC steps and Langevin dynamics lead us to combine the two. We now run the same simulation with alternating MC steps and Langevin dynamics, as described in the main text. Figure 3 shows three snapshots from this simulation run (see also S9 video). As expected, at $t = t_0$ the Langevin dynamics have completely relaxed the initial perturbation.

At later times, we see the MC steps again causing steps to other minima of $D(\Delta p)$.

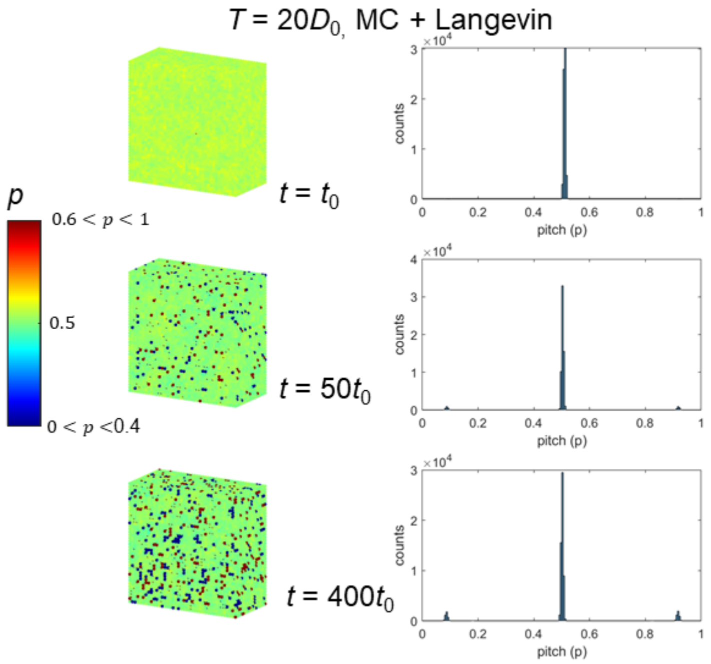

Figure 3: Simulation example: relaxation of a small perturbation at $T < T_c$ using both MC steps and Langevin dynamics. Three time points are shown. Left column shows the interior of the simulation volume with a restricted color scale to show detail (the color of sites with $p \approx 1/12$ and $11/12$ are oversaturated at the color scale limits). Right column shows histograms of all pitches in the lattice.

To compare the three methods of thermalization above, we plot the average dissonance $\overline{D}$ per site vs. time in Fig. 4. The initial state with the Gaussian perturbation has $\overline{D}$ close to the ground state of $\overline{D} = 0$. In the case of Langevin dynamics only (see inset to Fig. 4, we see rapid thermalization to a constant value of $\overline{D}$. At $T = 20D_0$ (red line), where correlations still play some role relatively close to T_c, $\overline{D}$ saturates to $\overline{D} \approx 24D_0$, somewhat greater than T. Farther from T_c, at $T = 5D_0$ (black line), $\overline{D}$ saturates to $\overline{D} \approx 5D_0$, as equipartition predicts for a one-dimensional harmonic oscillator.

In the main panel of Fig. 4, the orange line shows the run at $T = 20D_0$ with MC steps only. This curve starts near $\overline{D} = 0$, as the Langevin dynamics are not present to rapidly thermalize the initial perturbation. Instead, $\overline{D}$ grows slowly as the MC steps cause jumps to distant values of pitch, and slowly relaxes the initial perturbation. At $t = 400t_0$, the curve has still not saturated, in agreement

with the images above showing that the initial perturbation has not yet relaxed to thermal equilibrium at this time.

In contrast, the blue curve in Fig. 4 shows the run at $T = 20D_0$ with both MC steps and Langevin dynamics, as in the main text. Here, the Langevin dynamics cause very rapid thermalization within a single potential well at $t < t_0$, (not visible here, but similar to the inset.) So the blue curve already starts at $\overline{D} \approx 24D_0$. The subsequent increase in $\overline{D}$ is caused by the MC steps again exciting lattice sites to distant values of pitch. These excitations have largely reached equilibrium by $t = 400t_0$.

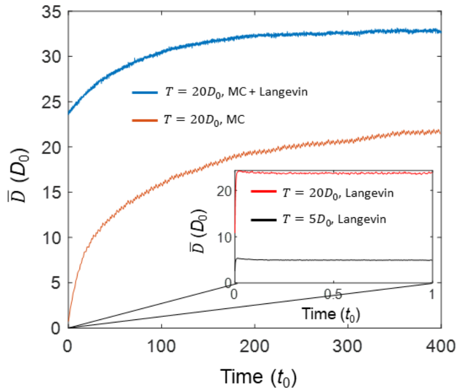

Figure 4: Average value $\overline{D}$ of total dissonance at all sites during relaxation from the perturbation described above and shown in Figs. 1-3. Inset shows a reduced time range. The red line in the inset corresponds to the run in Fig. 1, the orange line in the main figure to Fig. 2, and the blue line to Fig. 3. The black line in the inset is similar to the run in Fig. 1, but at lower $T = 5D_0$.

1.2 Thermalization above T_c

Here we test the simulation procedure starting from an initially perturbed state at $T > T_c$. Again, we will see roles of both the MC steps and the Langevin dynamics. Here we can also verify that the perturbation is relaxed by the propagation of a front, and that the speed of that front agrees roughly with the length- and time-scales given by the correlation length ξ and correlation time τ found in the main text.

At $T > T_c$ the equilibrium state is largely disordered. As such we initialize the pitches to uniform random numbers on the interval $[0, 1)$, which is then perturbed by multiplying the pitch at position (x, y, z) by

$$f(x, y, z) = 1 - \sin^2 \frac{\pi x}{40} \sin^2 \frac{\pi y}{40} \sin^2 \frac{\pi z}{40}. \tag{2}$$

The resulting lattice remains fully disordered at the edges, but the range of

the disorder reduces continuously to zero at the center of the cube. For clarity of plotting, we also shift all the pitches by 0.5 mod 1.

Starting from this initial state, we first run the simulation using the combination of MC steps and Langevin dynamics as in the main text at $T = 31T_0$ ($\varepsilon = 0.13$). Three snapshots are shown in Fig. 5 (see also S10 video). Again, the left shows the lattice at the indicated times, with only half the sites plotted so the perturbation in the interior is visible. The plots on the right show the histogram of pitches on the lattice. The first image at $t = 1t_0$ shows the perturbation as the green region in the middle. The histogram shows that the pitches have already begun to thermalize (mostly due to Langevin dynamics) into a nearly uniformly disordered region (nearly flat histogram) surrounding a region of nearly constant pitch (sharp peak near $p = 0.5$). In the second image at $t = 25t_0$ we see that the green region is still present, but with reduced size. The histogram shows that the disordered region is now largely uniform, with a smaller sharp peak (Which has also drifted slightly to lower pitch). Finally, at $t = 100t_0$ the perturbation has fully relaxed, and the lattice is entirely in the equilibrium disordered state.

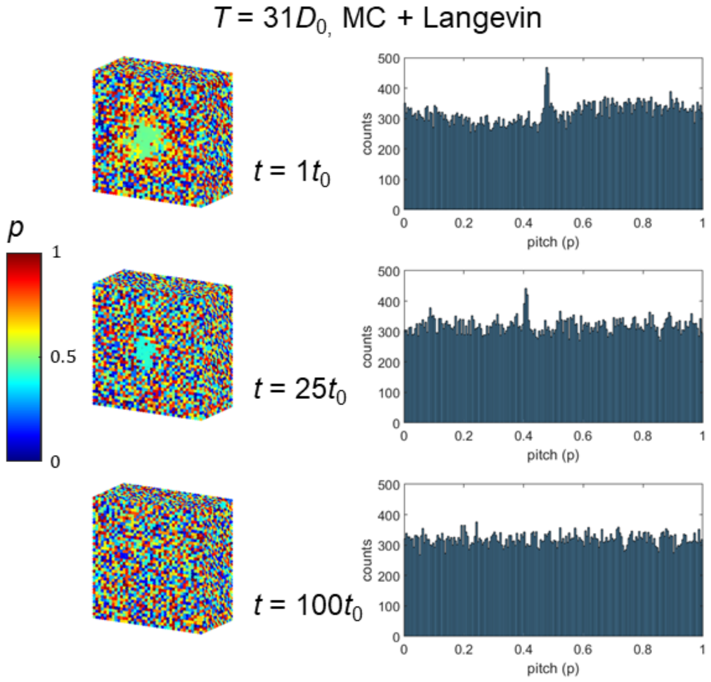

Figure 5: Simulation example: relaxation of a perturbation away from the disordered state at $T > T_c$ using both MC steps and Langevin dynamics. Three time points are shown. Left column shows the interior of the simulation volume. Right column shows histograms of all pitches in the lattice.

We next compare to the same simulation, but with Langevin dynamics only. The results are shown in Fig. 6 (see also S11 video). The first time point at $t = 1t_0$ looks quite similar to the case above with both MC steps and Langevin dynamics, as it is mainly the Langevin dynamics that have initially relaxed the lattice to a uniform random region surrounding a region of nearly constant pitch. As the simulation continues, however, we see that the region of constant pitch shrinks much more slowly, with the peak in the histogram only slightly reduced at $t = 100t_0$. This occurs because the region of nearly constant pitch is a low energy (dissonance) configuration with a large barrier separating from other relatively low-dissonance configurations. At this temperature, those slightly higher-dissonance configurations should be explored, but the Langevin dynamics are very inefficient at getting there.

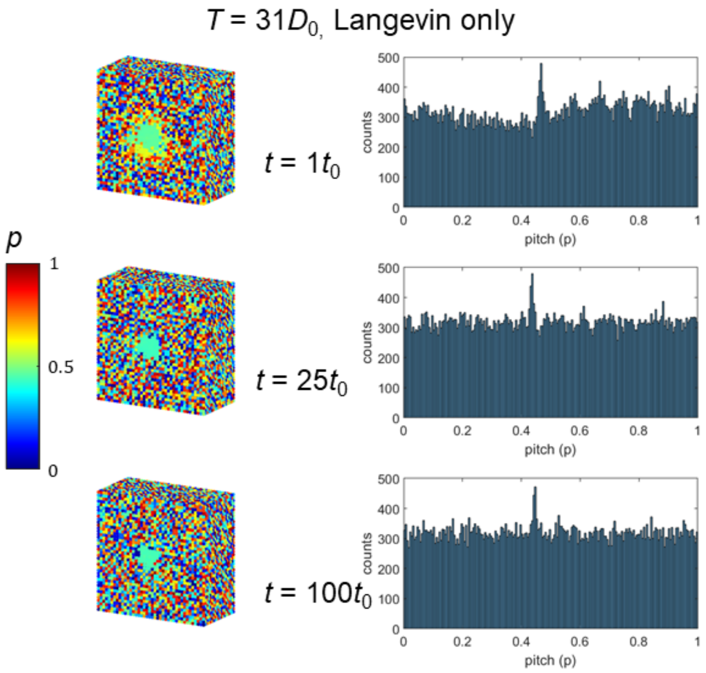

Figure 6: Simulation example: relaxation of a perturbation away from the disordered state at $T > T_c$ using Langevin dynamics only. Three time points are shown. Left column shows the interior of the simulation volume. Right column shows histograms of all pitches in the lattice.

On the other hand, we compare to the same simulation with the MC steps only. These results are shown in Fig. 7 (see also S12 video). In this case, the first time point at $t = 1t_0$ still clearly shows the initial artificial pitch distribution. The random uniform distribution is multiplied by the function $f(x, y, z)$ that increases the proportion of pitches near zero, at the expense of pitches near 1,

and then this distribution is shifted by 0.5 mod 1, resulting in the histogram as shown. In the other cases, the Langevin dynamics quickly smoothed out this unusual-looking distribution. Over time, we see that the MC steps decrease the amplitude of the perturbation on a similar timescale to the first case above with both MC and Langevin dynamics, but the pitch distribution retains the initial, artificially-imposed shape all the way to $t = 100t_0$. These cases demonstrate the different roles of the MC steps and Langevin dynamics in this temperature regime. The Langevin dynamics efficiently thermalize the state within the local potential minimum, but the thermalization of regions of distinctly different pitches (with pitches in different mimima of $D(\Delta p)$) requires MC steps. Therefore, it is predominantly the MC steps that are responsible for the propagation of a front that can thermalize regions of different pitch. These results also verify that regions of different pitch thermalize by propagating fronts, as opposed to randomization from isolated nucleation of MC switches. Though isolated MC switches do occur, the probability of nucleating and growing a new region within a region of constant pitch is low as compared to MC switches at an existing boundary between pitches causing propagation of that boundary.

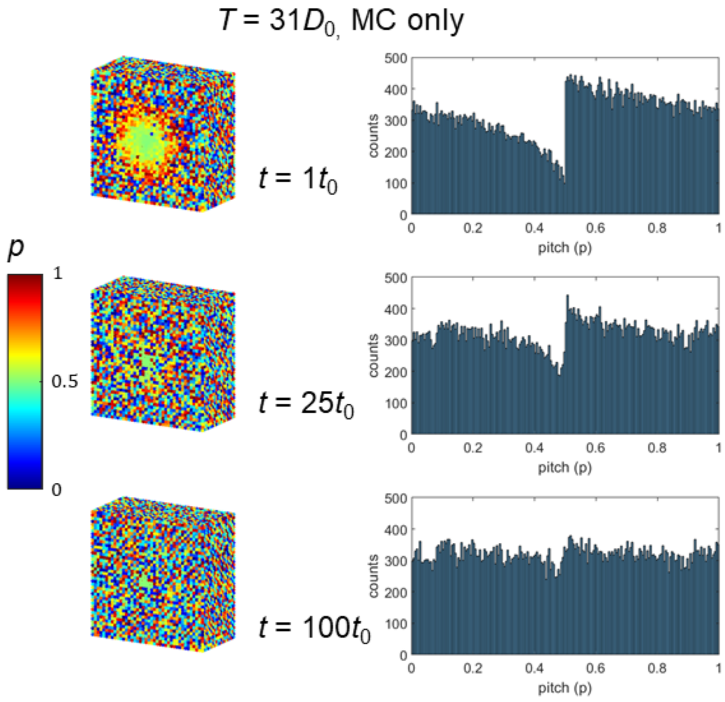

Figure 7: Simulation example: relaxation of a perturbation away from the disordered state at $T > T_c$ using MC steps only. Three time points are shown. Left column shows the interior of the simulation volume. Right column shows histograms of all pitches in the lattice.

We can compare the different cases above for thermalization at $T > T_c$ by plotting the maximum of the histograms shown in the figures. This is shown in Fig. 8. The three cases above are shown, as well as a run with MC steps and Langevin dynamics, but at $T = 30D_0$ ($\varepsilon = 0.09$). As expected, the case of Langevin dynamics only is taking much longer to thermalize. The case with MC steps only does not attain the same maximum, as the perturbed region does not form a sharp peak in the histogram, then falls off fairly rapidly, but fails to thermalize fully, as was seen in Fig. 7. Finally, the two cases with both MC steps and Langevin dynamics clearly thermalize in a continuous fashion as the perturbed region shrinks.

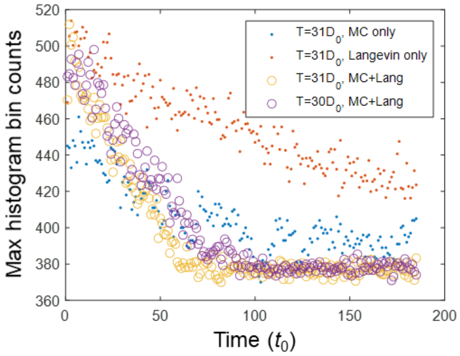

Figure 8: Relaxation of a perturbation at $T > T_c$ with different simulation methods. The maximum value of the histogram bins as shown in Figs. 5-7 is plotted vs. simulation time. The yellow circles correspond to the full simulation method at $T = 31\ D_0$ as shown in Fig. 5. The red dots correspond to Langevin dynamics only, as shown in Fig. 6, and the blue dots correspond to MC steps only, as shown in Fig. 7. The purple circles are similar to the yellow circles, but with a slightly lower $T = 30\ D_0$.

From Fig. 8, we can extract an approximate propagation speed for the encroachment of the disordered region into the perturbed region. The initial perturbation to the pitch lattice has a characteristic length of $l \approx 20a_0$, where a_0 is the lattice constant and l is set by the distance from maximum to minimum of the perturbation. At $T = 31D_0$, thermalization is complete in time $t \approx 60t_0$, and at $T = 30D_0$, thermalization is complete in time $t \approx 80t_0$. We can then obtain an approximate speed $v \approx l/t \approx 0.33a_0/t_0$ at $T = 31D_0$, and $v \approx 0.25a_0/t_0$ at $T = 30D_0$. We can compare this to the speed $v = \xi/\tau$, as found from the correlation length and time in the main text. From the equations given in the main text, we expect $v \approx (\xi_0/\tau_0)\varepsilon^{\nu}$ near the critical point. Using the fit results for the M_7 order parameter, we obtain $v \approx 1.7\ a_0/t_0$ at $T = 31\ D_0$ and $v = 1.4\ a_0/t_0$ at $T = 30\ D_0$. These two ways of determining the speed of fluctuations on the lattice agree within an order of magnitude. Better agreement is not to be expected. Because the speed is set by how well the MC steps can move

a boundary between pitches, the speed will depend on what the specific pitch difference is at boundary, as well as the shape of the boundary. Nonetheless, the agreement in order of magnitude yields evidence that the formation of vortices upon quenching is indeed caused by the lack of time for uncorrelated regions to thermalize, as predicted by the KZM.

2 Further characterization of the transition and critical behavior

2.1 Finite size scaling

Technically, phase transitions only occur in infinite systems. In a finite system, thermodynamic quantities to do not actually diverge, but instead peak at a finite value. By studying the behavior of these quantities as a function of system size L, we can extrapolate to the infinite system. Furthermore, due to the scaling behavior of the system near a critical point, we can use the trends vs. L to calculate the critical behavior quantitatively [1, 2]. In many cases, this approach yields very accurate values of critical exponents and critical points. Here, we find that the complexities of this model, such as the multiple order parameters, multiple transitions, possible weak first-order behavior, and the relatively computationally expensive simulation, yield plausible estimates of these quantities, though still with some uncertainty

Though many thermodynamic quantities are predicted to scale with L, since we have different transitions for different order parameters M_k, we should only look at quantities derived from M_k and not from the total energy. Specifically, we study how the susceptibility $\chi_k = \langle M_k - \langle M_k \rangle\rangle^2/T$ vs. ε changes with L. We simulate quenches at $\tau_Q = 32760t_0$ on an $L \times L \times L$ lattice, as described in the main text, with L varying from $L = 30$ to $L = 5$. At these values of L, the quench is sufficiently slow for the system to achieve equilibrium following the quench. During each temperature step, we calculate χ_k averaging over the duration of that step. Since it is difficult to completely achieve equilibrium at larger L, we also subtract a smoothed line (a LOESS filter with width $60t_0$) from M_k at each temperature step before calculating the variance to find χ_k. Since $60t_0$ is greater than the correlation time in the range studied here, this should not greatly affect the fluctuations used to calculate χ_k. Figure 9 shows plots of $|M_7|$ and χ_7 on the left, and $|M_{12}|$ and χ_{12} on the right for L ranging from 5 to 30. Values of χ_k are averaged over two repetitions to reduce noise. As expected, χ_k does not diverge, but instead shows a finite peak at an effective $T_c(L)$ that shifts with L. For accurate determination of χ it is important that the system achieves equilibrium in each temperature step. This precludes going to larger L, as τ_Q must be very large to stay in equilibrium through the transition (as seen in Fig. 11, which took several weeks to run.) Furthermore, the simulation should sample all of configuration space at each time step, which takes even longer. As it is, some points near T_c do not completely reach equilibrium, and if there is weak first-order behavior, we do not sample both bistable regions of configura-

tion space. That is, the quench behaves more like a hysteretic transition, than one with phase coexistence.

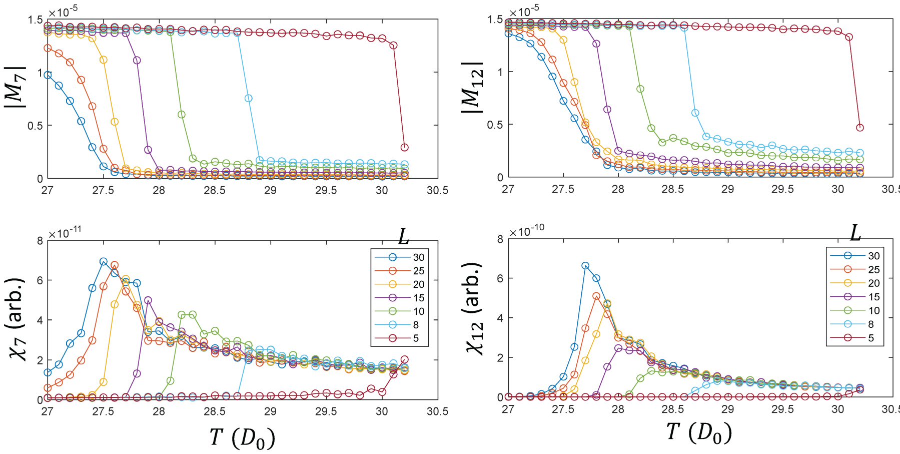

Figure 9: Finite size scaling. Parameters extracted from simulated quenches at $\tau_Q = 32760 t_0$ with lattice size $L = 5 - 30$. Top panels show two order parameters $|M_7|$ and $|M_{12}|$ with $T_c(L)$ clearly shifting to lower values as L is increased. Bottom panels show the corresponding susceptibilities χ_k extracted from fluctuations of the order parameters, with $T_c(L)$ deinfed as the peak of each curve. Bottom panels are the average of two runs.

Nonetheless, we can extract values of $T_c(L)$ as the location of the maxima of χ_k. Finite size scaling theory predicts that these values should scale linearly with $L^{-1/\nu}$, and should intercept the y-axis at the true critical value of $T_c(\infty)$. We perform a three-parameter fit to the function $T_c(L) = mL^{-1/\nu} + T_c(\infty)$, with parameters m, ν, and $T_c(\infty)$. The fit results are shown in Fig. 10 and summarized in Table 1 below. We see that the values of $T_c(\infty) = (27.3 \pm 0.1)D_0$ for $k = 7$ and $T_c(\infty) = (27.6 \pm 0.1)D_0$ for $k = 12$ agree reasonably well with values estimated from the plots of M_k vs. T above. We also see that the values of $\nu = 0.66 \pm 0.06$ for $k = 7$ and $\nu = 0.60 \pm 0.08$ for $k = 12$ are systematically higher than those obtained from fitting ξ vs. ε. This agrees with hypothesis that those values of ν were too small due to the difficulty of fitting values of $\xi < 1$ and the large scatter in points at larger ε. These values of ν are in the vicinity of those expected for the 3D XY model ($\nu = 0.66$), and agree less well with the 3D 3-state Potts model ($\nu = 0.5$), but the uncertainty is still too large to conclusively determine between them. The values from finite-size scaling near $\nu = 0.66$ would also yield a value of $z \approx 2$ given the values for $z\nu$ obtained from fitting to τ vs. ϵ.

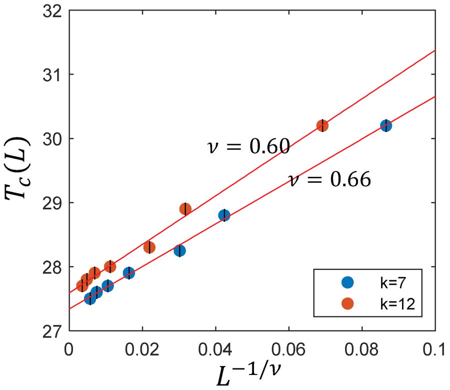

Figure 10: Critical exponents from finite size scaling. A three-parameter fit to $T_c(L)$ extracted above for the $k = 7$ and $k = 12$ order parameters vs. $L^{-1/\nu}$, yielding the labeled values of ν. Error bars indicate temperature resolution of the simulation runs used here.

2.2 Additional characterization of the transition

If we zoom in on the order parameters M_k near T_c, we can study the detailed form of the transition. Figure 11(a) shows $|M_k|$ for $k = 1 - 20$ during a slow quench through T_c, with $\tau_Q = 2.73 \times 10^5\ t_0$ (see also S13 video). After the transition, at $T = 20\ D_0$, τ_Q is decreased substantially to finish the simulation more quickly. In this quench, we see that all $|M_k|$ approach 1 at low T, the expected ground state. Even if the relaxation time and correlation length do indeed diverge at T_c, we can still relax to the ground state because the system here is finite.

The inset to Figure 11(a) shows the region around T_c in more detail. Here we see a range of $\approx \pm 0.2\ D_0$ where fluctuations in M_k become large enough that it is difficult to precisely identify T_c. However, even beyond the initial fluctuations, M_{12} appears to increase at higher T than the other M_k. This is consistent with the mean field results in Ref. [1]. In that work, a slightly different $D(\Delta p)$ was used in which the critical band width was taken to be same for all partials. In the mean field approximation, two well-separated phase transitions were observed. First, the M_{12} order parameter undergoes a transition and all other M_k stay at zero. This single-mode transition is understood from Landau theory in Ref. [1]. Once M_{12} becomes large enough, coupling between modes enables the transition for the rest of the M_k. With the function $D(\Delta p)$ used here, and in a 3D lattice instead of the mean field approximation, we find a smaller separation between the two transitions, but the separation does appear to be present.

We also perform a slower temperature sweep up over a small range, from $T = 27\ D_0$ to $30\ D_0$ at $\tau_{up} = 2.73 \times 10^5\ t_0$, shown in Fig. 11(b). This simulation is initialized to the state of the system at $T = 27D_0$ from the quench in Fig. 11(a). We again see hysteresis as compared to the quench down in (a), with $T_c =$

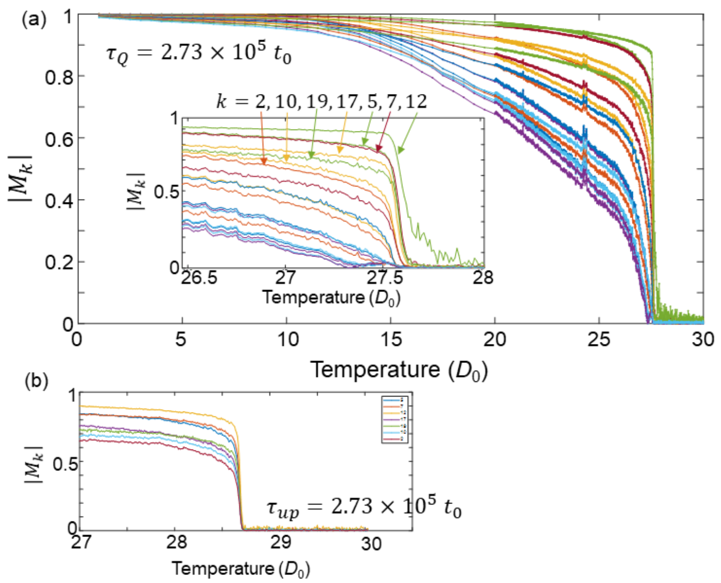

Figure 11: Slow quench and ramp up across T_c. (a) Magnitude of order parameters $|M_k|$ ($k = 1 - 20$) for a quench at $\tau_Q = 2.73 \times 10^5\ t_0$. Small glitches such as near $t = 24\ D_0$ occur due to small numerical error in the periodic boundary conditions. Inset shows a zoomed-in region of the same data with prominent values of k labeled. (b) Magnitude of order parameters $|M_k|$ for a temperature ramp up at the same rate as (a), starting from the point at $T = 27\ D_0$ from (a).

$28.7\ D_0$. The presence of hysteresis in this slow ramp suggests that it is not an artifact of the finite ramp time. Furthermore, in the sweep up, there appears to be little to no difference in T_c between $k = 12$ and the other order parameters. This is also understandable in the mode coupling picture. Coming from below T_c, all M_k are non-zero and the coupling between these modes pushes up T_c for all modes.

From the above discussion, we see that the nature of the transition in this system has several complications. The hysteresis is suggestive of a first order transition, but there is no clear discontinuity in the order parameters, and the correlation length and relaxation time appear to be diverging as a power law up to T_c, suggestive of a second order transition. It seems plausible that this transition is weakly first order, meaning that the bistable configurations are only present over a small range, and are close to each other. In such a case, the critical behavior can still appear second-order-like. The three-state, 3D Potts model is an example of a weakly first order transition [3, 4]. In the three-state Potts model, each site takes one of three values. If interacting sites are in the same state, the energy is low, and if they are in different states, the energy is high. In three dimensions, it has been shown that this model undergoes a first-order transition, but with only a small discontinuity, and second-order-like

critical behavior. There is some reason to think we might see similar behavior here. While at high energy, our model is a continuous XY model, at lower energy, sites are most likely to fall into one of the minima of the potential $D(\Delta p)$. In $D(\Delta p)$, there is one global minima, and two degenerate next-deepest minima. If the interactions are mainly near those three minima, then the situation is similar to the 3-state Potts model. The situation here is somewhat different though, in that the three minima are not equidistant in Δp so the sites do not take on just three values. Because the two second-deepest minima are near $\Delta p = 5/12$ and $\Delta p = 7/12$, the sites instead take on 12 values. These questions may provide interesting topics for further study.

Even though the relaxation time does not truly diverge in a first order transition, if the subcriticality is weak, the relaxation time can still grow large enough to freeze in defects during a quench, as predicted by the KZM [5, 6]. In any case, the facts that 1) we empirically observe the expected power law behavior of the relaxation time and correlation length quite close to T_c (Fig. 4 in the main text), and 2) we observe frozen-in defects in accordance with the KZM suggest that any subcriticality is not strong enough to wash out the KZM here.

The fluctuations in the order parameters around T_c seen in Fig. 11(a) raise the question of which features are repeatable, and which are random fluctuations. To address this question, we plot $|M_{12}|$ and $|M_7|$ for the same scan repeated 10 times, in Fig. 12. Each scan is a quench at $\tau_Q = 16380\ t_0$, and is the data set used for the measurement of τ in the main text. Each curve is normalized to its value at $T = 25D_0$ for easier comparison. Here we can see that the separation between M_{12} and M_7 is consistent across repeated runs. We also see that there are visible fluctuations that sometimes occur within a range of $\approx 0.2D_0$ before the consistent rise in the order parameter. To estimate the value of T_c for the fitting in the main text, we extrapolate the rise in $|M_k|$ down to zero, assuming the ideal curve has a sharp kink or jump at T_c. From this procedure we estimate $T_c = 27.85D_0$ for $k = 12$ and $T_c = 27.3D_0$ for $k = 7$ (and all other k, not shown).

2.3 Zooming in on the frozen region

In Fig. 5(b) of the main text, the frozen region $\varepsilon = \pm\hat{\varepsilon}$ is too small to make the behavior of ξ vs. ε in this region visible. Here we show results from runs with finer steps to examine this region more closely. As ε approaches zero from above during a quench, we first expect ξ to increase as a power law. We also expect fluctuations in ξ to increase, as well as the relaxation time τ. Once the time remaining to reach $\varepsilon = 0$ is less than the relaxation time τ, the system will no longer be able to relax to equilibrium and we expect to see a flattening of ξ away from the power law increase in this region. This is the prediction of the KZM as described in the main text, where this frozen region occurs in the range $\varepsilon = \pm\hat{\varepsilon}$. Once $\varepsilon < -\hat{\varepsilon}$ (assuming symmetric behavior about $\varepsilon = 0$), then the system can adiabatically relax, though will generally not be able to achieve equilibrium because topological defects will remain frozen in, even as T goes to zero.

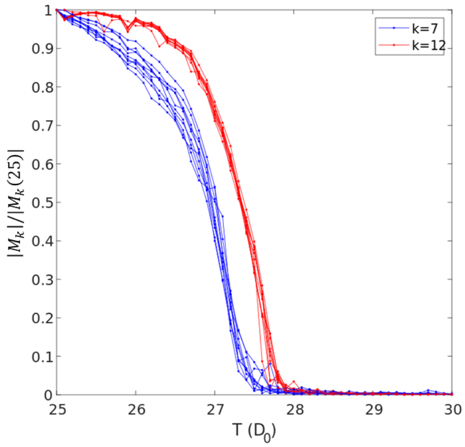

Figure 12: Order parameters $|M_k|$ ($k = 7, 12$) normalized by their values at $T = 25\ D_0$ for 10 repetitions of the same quench at $\tau_Q = 16380\ t_0$.

Figure 13(a) shows the results for the correlation length ξ_7 (for order parameter M_7) vs. ε at two different τ_Q. These results are obtained by performing repeated simulation runs, extracting ξ_7 from each run, and averaging them together. The results for $\tau_Q = 3276\ t_0$ are the average of 11 runs, and the results for $\tau_Q = 1638\ t_0$ are the average of 15 runs. Near $\varepsilon = 0$, the fluctuations in ξ become large, with a correlation time persisting over the range of $\hat{\varepsilon}$ by definition, making averaging particularly essential here. The thick bars in Fig. 13(a) show the expected value of $\hat{\xi}$ and the expected frozen range $\pm\hat{\varepsilon}$, as calculated from the fitted values of τ_0, ξ_0 ν, and $z\nu$. As was discussed in the main text, the expected values of $\hat{\xi}$ approximate the observed values, and we can see an inflection in the curve of ξ_7 over approximately the expected range of ε. Instead of a divergence at $\varepsilon = 0$, or a continuously increasing slope of ξ as ε decreases, we can see a flattening in the $\pm\hat{\varepsilon}$ region.

For comparison, we sketch the expected shape of ξ vs. ε in Fig. 13(b) at these two values of τ_Q. The initial power law increase as ε decreases is calculated directly from the fitted values of τ_0, ξ_0, ν, and $z\nu$. Likewise, the frozen range $\pm\hat{\varepsilon}$ is also calculated. Within the frozen range, ξ is shown to increase more slowly, as is predicted for the KZM [7]. At $\varepsilon < -\hat{\varepsilon}$, ξ begins to increase more rapidly again, as the system can adiabatically relax. The particular amount of the increase in the frozen and adiabatic regions, as well as the particular shape of the curve are just sketched here, but serve to show plausible agreement with the results in Fig. 13(a).

2.4 Other order parameters

In the main text, we focus on the critical behavior of the order parameter M_7. While $|M_{12}|$ has the largest value, the fact that it saturates to $|M_{12}| = 1$ in

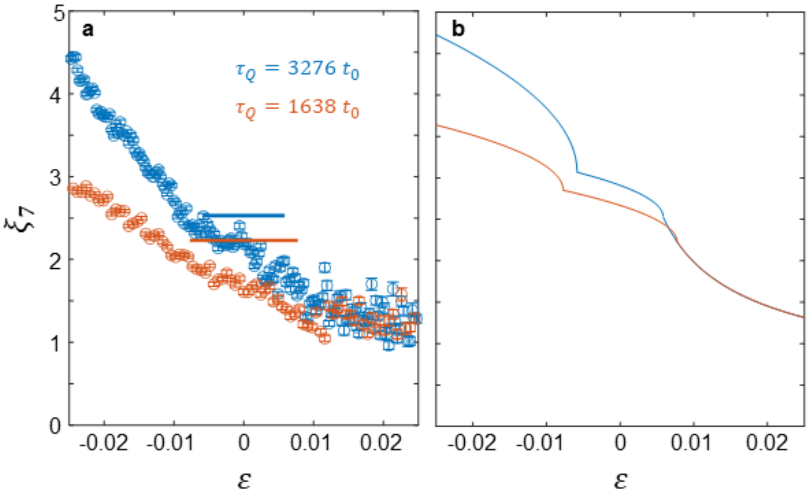

Figure 13: Zoom-in on the freeze-out region. (a) Correlation length ξ_7 for order parameter M_7 in a small region around $\varepsilon = 0$, for two values of τ_Q. The bars indicate the predicted frozen correlation length $\hat{\xi}$ over a range $\varepsilon = \pm\hat{\varepsilon}$ for both τ_Q. Data for $\tau_Q = 3276\ t_0$ are the average of 11 runs, and data for $\tau_Q = 1638\ t_0$ are the average of 15 runs.

all quenches demonstrates that the defects (vortices) that form in the 12-fold order of the quenched state are not frozen in as the temperature approaches zero. That is, the low energy vortices maintain 12-fold order. For example, a vortex (corresponding to a major triad) may form with pitches close to $p = 0$, $p = 4/12$, and $p = 7/12$. These three pitches all yield the same value of $e^{2\pi i 12 p}$, but not the same value of $e^{2\pi i 7 p}$. As such, we should look at an order parameter like M_7 observe the arrangement of defects at low temperature. However, we can still study the critical behavior for M_{12} and other order parameters.

Figure 14 shows the same analysis of τ and ξ vs. ε as shown in Fig. 4 of the main text, but here for M_{12}. We use the value obtained above of $T_c = 27.85 D_0$ for this analysis. We obtain similar results for the critical exponents, and scale factors as we did for the case of M_7. Figure 14a shows results for τ vs. ε on a log-log scale. Figure 14b shows results for ξ vs. ε on a log-log scale. Values of τ and ξ are obtained and fit in the same way as in the main text from the same dataset, but in this case for the order parameter M_{12}. As in the main text, we fit the log-log data to a straight line. The fit parameters are given in the caption to Fig. 14. In general, the results for all fit parameters match the order of magnitude for the $k = 7$ case. As for $k = 7$, the exponents lie reasonably close to well-studied related systems such as the 3D XY model ($\nu = 0.66$, $z\nu = 1.33$) or the 3D 3-state Potts model ($\nu = 0.5$, $z\nu = 1.0$).

Table 1 shows the results of the same analysis for several of the prominent M_k. We see that τ_0 and ξ_0 are of the same order of magnitude for these values

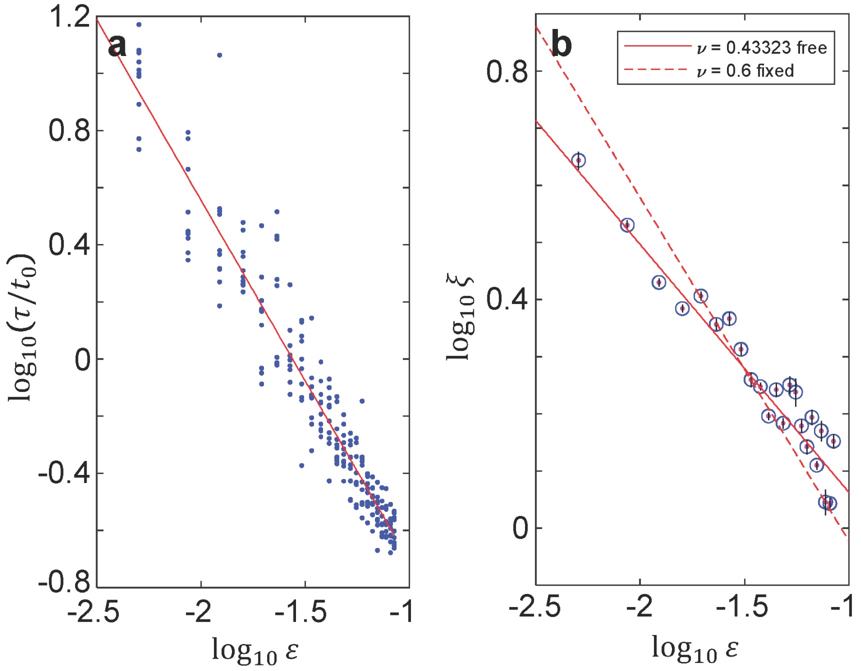

Figure 14: Fitting critical behavior of the M_{12} order parameter. Fit parameters are as follows. (a) $\tau_0 = (1.1 \pm 0.2) \times 10^{-2} t_0$, $z\nu = 1.27 \pm 0.06$. (b) Solid line: $\xi_0 = 0.43 \pm 0.04$, $\nu = 0.43 \pm 0.02$ (ν varied in fit), and Dashed line: $\xi_0 = 0.24 \pm 0.01$, ($\nu = 0.60$ fixed from finite size scaling).

of k, and that the exponents tend to cluster near values for similar systems.

With the critical behavior fitted for the M_{12} order parameter, we can also compare to the prediction of KZM for this order parameter. While we do not observe vortices frozen into the 12-fold order at low temperature, we can still see pause in growth of $\hat{\xi}$ in the frozen region in the range $\pm\hat{\varepsilon}$. Figure 15(a) shows the values of ξ obtained in the same way as in Fig. 13(a), but using the M_{12} order parameter. Again, as ε decreases we first see a power law increase. Though perhaps less pronounced here, an inflection in the curve can be seen near $\varepsilon = 0$. The red bar in Fig. 15(a) indicates the calculated value of $\hat{\xi}$ and range $\pm\hat{\varepsilon}$ based on the fit results of Fig. 14. Then at negative ε we see a very rapid rise in ξ. Though not shown here, ξ rapidly becomes comparable to, or larger than, the system size. (These results are not shown as the fitting becomes unreliable in this case.) This rapid growth of ξ in the adiabatic relaxation regime may be responsible for the lack of defects in the M_{12} order parameter at low temperature. The expected spacing of the frozen-in defects is large compared to the system here, so instead we observe uniform 12-fold order across the finite

	fit		fit		FSS	
k	$z\nu$	τ_0 $(10^{-2}t_0)$	ν	ξ_0	ν	ξ_0
5	1.38	1.0	0.53	0.15		
7	1.39	1.1	0.45	0.25	0.66	0.10
12	1.27	1.1	0.43	0.43	0.60	0.24

Table 1: Fit results for critical exponents and scale factors for three different order parameters M_k. The second through fifth columns are obtained from direct fitting to ξ and τ vs. ε. The second to last column is obtained from the finite size scaling described above, with the final column obtained by fitting ξ vs. ε with the fixed value of ν in the previous column.

system.

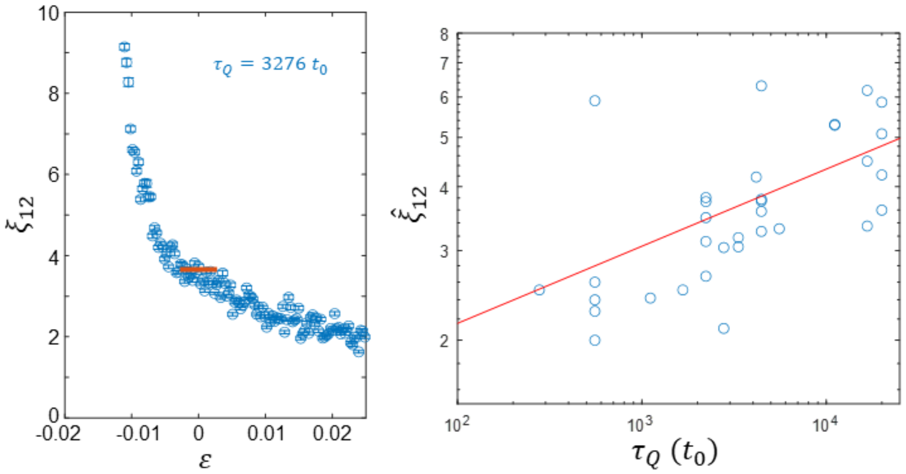

Figure 15: Signatures of KZM for the M_{12} order parameter. (a) ξ vs. ε in a small range about $\varepsilon = 0$ from the average of 11 quenches at $\tau_Q = 3276\ t_0$. Red line indicates predicted value of $\hat{\xi}$ and range $\pm\hat{\varepsilon}$. (b) Data points show measured values of $\hat{\xi}$ from simulation runs at different τ_Q. Red line shows predicted $\hat{\xi}$ vs. τ_Q based on the fit results shown in Fig. 14.

3 Repeatability of the results

In the main text, we showed results from particular runs at different τ_Q. To demonstrate repeatability, and to give some sense of how the results change with τ_Q, here we present additional results. Figure 2 in the main text shows the simulation results for a quench at $\tau_Q = 3276\ t_0$ with the quenched state

at $T = 1D_0$ displaying domains of 12 distinct pitches. In Fig. 16 we show the quenched state of two additional runs at $\tau_Q = 13650\ t_0$ and $\tau_Q = 546\ t_0$ using the same color scales as in the main text. Videos of these quenches are also provided in S1 video, S3 video, and S5 video. As was also seen in the main text in Fig. 5, the slower quench produces larger pitch domains and the faster quench produces smaller pitch domains. As can be seen in the corresponding histograms of pitches, both cases also have 12 distinct pitches. And finally, we plot the vortex strings for both cases. As expected, the larger pitch domains have a sparser network of strings and the smaller pitch domains have a denser network of vortex strings. Videos of the formation of the vortex strings during the quench are provided in S2 video, S4 video, and S6 video.

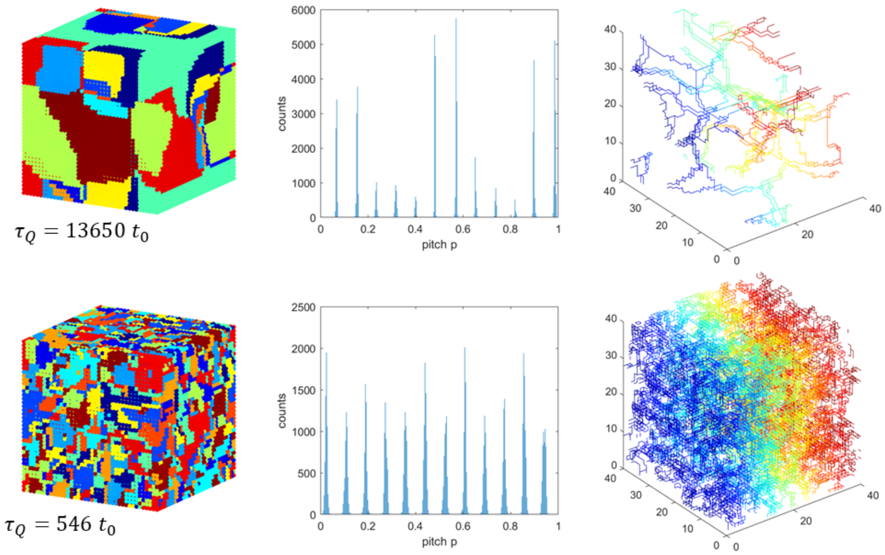

Figure 16: Quenched state of the simulation at two different values of τ_Q. The top row corresponds to $\tau_Q = 13650\ t_0$ at the final temperature $T = 0.1\ D_0$, and the bottom row corresponds to $\tau_Q = 546\ t_0$ at the final $T = 1\ D_0$. Each row shows, from left to right, the final state of the lattice, a histogram of all pitches in the lattice, and the vortex strings in that state.

The characteristics of the general arrangement of pitches is also quite repeatable across different simulation runs, and at different τ_Q. Table 2 lists statistics illustrating how the pitches are arranged in four different runs. (The run at $\tau_Q = 4368t_0$ is discussed in Fig. 6 in the main text, and the run at $\tau_Q = 1638t_0$ is discussed in Fig. 7 in the main text.)

The second and third columns of Table 2 display the number of consonant nearest neighbors, and the number of dissonant nearest neighbors present in the lattice. Consonant neighbors are taken to be p4, p5, m3, M3, m6, and M6 intervals, and dissonant neighbors are taken to be m2, M2, tritone, m7, and

M7 intervals. In all cases, consonant neighbors greatly outnumber dissonant neighbors. As expected, the total number of intervals decreases with increasing τ_Q as the pitch domains are increasing in size.

τ_Q (t_0)	# cons.	# diss.	% triads cont.	% triads closed	% tetrads cont.	% tetrads closed
546	67224	3983	94	59	99	65
1638	36057	1376	90	53	99.8	58
4368	25020	385	90	49	99	59
13650	20680	557	88	54	95	32

Table 2: Statistics of consonant and dissonant arrangements of pitches in the quenched state for four runs with different τ_Q.

We also verify the repeatability of the types of chords that comprise the vortices in the quenched state. Table 2 summarizes statistics of intervals and chords for the same four runs as in Table 1. As described in the main text, chords may be triads, in which three distinct pitches surround a point, or tetrads, where four distinct pitches surround a point. It is highly likely that the pitches of triads or tetrads form a continuous path on the Tonnetz (see Fig. 6(a) in the main text). This is because the connections on the Tonnetz represent the consonant intervals (p4, p5, m3, M3, m6, and M6), and we saw above that the large majority of intervals present in the quenched state are just these intervals. A chord will form a continuous path on the Tonnetz if at most one interval around the vortex is dissonant (m2, M2, tritone, m7, or M7). Furthermore, we see that often a majority of chords form a closed path on the Tonnetz. In these cases, all intervals around the vortex are consonant. Interestingly, we see that the fastest quench ($\tau_Q = 546\ t_0$) has some of the highest fractions of continuous and closed path chords. It is possible the higher density of vortices at the faster quench rate allows the vortices to more effectively relax to their lower energy (lower dissonance) configurations.

τ_Q (t_0)	% 2 or 3 pitches conserved	% new pitches adjacent
546	94	91
1638	96	91
4368	95	90
13650	95	87

Table 3: Statistics of the junctions of vortex strings in the quenched state for four runs with different τ_Q.

Finally, we verify the repeatability of the types of chord progressions that occur at junctions of vortex strings. In the main text, we argued that these

progressions are similar to the transformations between chords on the Tonnetz, as described by neo-Riemannian theory. We showed above that pitches around a vortex very frequently form continuous path on the Tonnetz, and often form a closed path. When a vortex string branches, we expect the neighboring chords to share some of the same pitches, and also expect the pitches unique to either chord to neighbor the shared pitches on the Tonnetz. Table 3 shows how often these conditions hold at junctions between vortex strings for the same four runs as in the previous tables. The second column shows the fraction of string junctions at which two or three pitches are shared between the chords on either side of the junction. At most three pitches can be conserved, because there can be at most four pitches around a vortex and at least one of them must change to have a junction. So we see that it is quite uncommon for only one pitch to remain constant across a junction, and it is even less likely to have no pitches in common (this is not shown in the table). The third column shows the fraction of junctions in which the changed pitches on either side of the junction are adjacent to the shared pitches, on the Tonnetz. We see that this percentage is also quite high. When the new pitches are not adjacent to the shared pitches, it is often the case that two new pitches have been added that are adjacent to each other, but only one is adjacent to the conserved pitches. The above discussion serves to show that conclusions of the manuscript remain valid across multiple runs, with different τ_Q and that the emergent features of musical harmony that we observe are robust.

References

[1] Fisher ME, Barber MN. Scaling Theory for Finite-Size Effects in the Critical Region. Phys Rev Lett. 1972;28:1516–1519. doi:10.1103/PhysRevLett.28.1516.

[2] Privman V. Finite size scaling and numerical simulation of statistical systems. World Scientific; 1990.

[3] Wu FY. The Potts model. Rev Mod Phys. 1982;54:235–268. doi:10.1103/RevModPhys.54.235.

[4] Herrmann H. Monte Carlo simulation of the three-dimensional Potts model. Zeitschrift für Physik B Condensed Matter. 1979;35(2):171–175.

[5] Casado S, González-Viñas W, Mancini H. Testing the Kibble-Zurek mechanism in Rayleigh-Bénard convection. Phys Rev E. 2006;74:047101. doi:10.1103/PhysRevE.74.047101.

[6] Miranda MA, et al. The Kibble-Zurek mechanism in a subcritical bifurcation. Phys: Condens Matter. 2013;25(40). doi:10.1088/0953-8984/25/40/404208.

[7] Biroli G, Cugliandolo LF, Sicilia A. Kibble-Zurek mechanism and infinitely slow annealing through critical points. Physical Review E. 2010;81(5):050101.
